# High resolution molecular and histological analysis of renal disease progression in ZSF1 fa/fa^CP^ rats, a model of type 2 diabetic nephropathy

**DOI:** 10.1371/journal.pone.0181861

**Published:** 2017-07-26

**Authors:** Ken Dower, Shanrong Zhao, Franklin J. Schlerman, Leigh Savary, Gabriela Campanholle, Bryce G. Johnson, Li Xi, Vuong Nguyen, Yutian Zhan, Matthew P. Lech, Ju Wang, Qing Nie, Morten A. Karsdal, Federica Genovese, Germaine Boucher, Thomas P. Brown, Baohong Zhang, Bruce L. Homer, Robert V. Martinez

**Affiliations:** 1 Inflammation and Immunology, Pfizer Worldwide Research and Development, Cambridge, Massachusetts, United States of America; 2 Clinical Bioinformatics, Early Clinical Development, Pfizer Worldwide Research and Development, Cambridge, Massachusetts, United States of America; 3 Drug Safety, Pfizer Worldwide Research and Development, Andover, Massachusetts, United States of America; 4 Nordic Bioscience A/S, Herlev, Denmark; 5 Drug Safety, Pfizer Worldwide Research and Development, Groton, Connecticut, United States of America; University Medical Center Utrecht, NETHERLANDS

## Abstract

ZSF1 rats exhibit spontaneous nephropathy secondary to obesity, hypertension, and diabetes, and have gained interest as a model system with potentially high translational value to progressive human disease. To thoroughly characterize this model, and to better understand how closely it recapitulates human disease, we performed a high resolution longitudinal analysis of renal disease progression in ZSF1 rats spanning from early disease to end stage renal disease. Analyses included metabolic endpoints, renal histology and ultrastructure, evaluation of a urinary biomarker of fibrosis, and transcriptome analysis of glomerular-enriched tissue over the course of disease. Our findings support the translational value of the ZSF1 rat model, and are provided here to assist researchers in the determination of the model’s suitability for testing a particular mechanism of interest, the design of therapeutic intervention studies, and the identification of new targets and biomarkers for type 2 diabetic nephropathy.

## Introduction

Diabetic nephropathy (DN) is a leading cause of morbidity and mortality in patients with diabetes and accounts for 30–40% of all end stage renal disease (ESRD) in the US [1]. Current treatments for DN focus on dietary modifications, blood glucose control, and blood pressure control; however, a significant number of patients progress to ESRD and will require renal replacement therapy in the form of dialysis or transplant [2]. Medicare expenditures for ESRD were an estimated $31 billion in 2013, accounting for over 7% of Medicare-paid claims costs [3]. Given the increasing incidence of obesity and diabetes worldwide, a clear and present need exists for novel, transformative therapies that target the underlying pathobiological mechanisms of DN and its progression to ESRD [4].

Central to this ongoing effort is the characterization and use of preclinical models that recapitulate human disease. Indeed, many mechanistic insights have been gained through the study of available DN models. Nonetheless, these efforts are hampered, to some extent, by the limited number of models that faithfully reproduce features of both early and late human disease. Commonly used models of DN include Streptozotocin (STZ), a model of type I diabetes, and models of type 2 diabetes that use animals with leptin deficiency (ob/ob mice) or leptin receptor deficiency (db/db mice, Zucker Diabetic Fatty rats). In these leptin models, hyperphagy-induced obesity results in diabetes and renal complications that mimic early- to moderate-stage human DN [5]. These commonly used models do not, however, progress to advanced DN and ESRD, indicating that additional factors such as hypertension may be required for full disease progression [6, 7]. Consequently, efforts to elicit more aggressive disease in the diabetic setting have been described, for example by introducing hypertension through deletion of nitric oxide synthase (*eNOS*^-/-^) or insertion of an inducible renin gene (*Cyp1a1mRen2*), or the use of a naturally insulin resistant mouse strain such as BTBR (STZ *eNOS*^-/-^ or db/db *eNOS*^-/-^, STZ *Cyp1a1mRen2*, BTBR ob/ob; reviewed in [5, 6]).

One outcome of such efforts is the ZSF1 rat model, first described by Tofovic *et al* in 2000 [8]. ZSF1 rats are the F1 progeny of two strains that are heterozygous for leptin receptor mutations, lean female Zucker Diabetic Fatty rats (ZDF, +/fa) and male spontaneously hypertensive heart failure rats (SHHF, +/fa^cp^). The progeny of this cross become either lean or obese depending on whether they are heterozygous or homozygous for leptin receptor deficiency. Lean animals, while technically not lean, do not develop diabetes and diabetic complications like their obese littermates. Importantly, unlike ZDF rats, both lean and obese ZSF1 rats are hypertensive as they inherit genes for spontaneous hypertension [8, 9]. As a result, and unlike the parental backgrounds from which they are derived, obese ZSF1 rats develop progressively worsening renal disease that culminates in death at around 45–50 weeks of age with signs of ESRD [8, 10–13].

The obese ZSF1 rat is one of relatively few rodent models that mimic the natural history of progressive human type 2 DN. We therefore undertook a high resolution longitudinal analysis of age-matched lean and obese ZSF1 rats spanning from early DN through fulminant DN to ESRD in obese animals. To our knowledge, this is among the most comprehensive studies of renal disease progression in this model to date. To complement our histological assessment of fibrosis, we provide evidence that levels of Collagen type III breakdown product in the urine are a non-invasive biomarker of renal fibrosis and functional decline in ZSF1 rats. In addition, we present ultrastructural findings of renal disease. Finally, we identify transcriptome changes in glomerular-enriched tissue over time, and report statistically meaningful concordance between week 34 onwards ZSF1 rats and a published dataset of glomerular gene expression changes in human disease. Our data support the conclusion that the obese ZSF1 rat is a relevant pre-clinical model for the study of renal function decline to ESRD in the setting of type 2 diabetes.

## Materials and methods

### Animals

All procedures involving animals were reviewed and approved by the Pfizer Institutional Animal Care and Use Committee. Lean and obese ZSF1 littermates were obtained from Charles River Laboratories at 8 weeks of age (strain codes #379 and #378, respectively). A total of 60 male rats, 30 lean and 30 obese, were enrolled in the study. Animals were housed and acclimated under 12 hour light-dark cycles with free access to water and Purina 5008 chow. 24 hour urine samples were collected from animals in metabolic cages prior to the termination of each cohort at 12, 20, 24, 29, 34, and 41 weeks of age. Between 34 and 41 weeks of age, one lean animal was euthanized for hematuria, and two obese animals were found dead of unknown cause with no outward clinical signs. Animals were sacrificed by CO_2_ asphyxiation, and blood was collected via cardiac puncture. Kidney sections were immersion fixed for slide preparation, stored on ice for isolation of glomerular-enriched tissue, or snap frozen in liquid nitrogen for additional analyses.

### Clinical chemistry methods

Chemistry samples were analyzed on a Siemens Advia 1800 automated clinical chemistry analyzer using both colorimetric and immunoturbidimetric assays. Serum insulin levels were determined using the Mouse/Rat insulin kit (Mesoscale Discovery, K152BZC). NGAL and Kim-1 levels were determined using the Kidney Injury Panel-1 Rat Kit (Mesoscale Discovery, K15162C).

### Tissue collection, slide preparation and staining

At necropsy, coronal sections of kidney were collected in a systematic unbiased approach from either the left or right kidney for immersion in 10% neutral buffered formalin and histopathology assessment. Paraffin-embedded kidney was sectioned serially at 4 mm, and sections were stained with H & E, periodic acid–Schiff (PAS) reagent with a hematoxylin counterstain, or with Masson Trichrome.

### Immunohistochemistry (IHC)

#### Staining for Collagen type IV

Immunohistochemical staining was performed on a DISCOVERY XT® automated stainer from Ventana Medical Systems (VMS). Sections were deparaffinized using EZ-prep (VMS). Antigen retrieval was applied using a Ribo CC buffer at mild setting (CC2 pH 6.0, VMS), followed by Protease 2 (VMS) enzyme digestion for 4 minutes. The primary, rabbit anti- Collagen type IV antibody (Thermo Scientific, Cat# PA1-26148) was applied at 10 μg/ml (1:300 dilution of a starting concentration of 3 mg/ml) for 60 minutes followed by DISCOVERY OmniMap anti-Rb HRP (VMS) for 16 minutes, and detection was completed in combination with DISCOVERY ChromoMap DAB Kit (VMS). Slides were counterstained with hematoxylin (VMS) for 12 minutes, followed by bluing reagent (VMS) for 12 minutes. After removal from the stainer, slides were washed in warm soapy water before being dehydrated through graded alcohols, cleared in xylene, and cover-slipped with synthetic mounting media.

#### Quantitative analysis of mean Collagen type IV staining area per glomerular tuft

Whole-slide images of the immunostained sections were obtained with Aperio AT2 whole slide scanner (Leica Microsystems GmbH). Glomerular tufts were manually selected by a systematic unbiased approach in every other quadrat for image analysis with Definiens Tissue Studio (Definiens AG). An average of 47 tufts was selected per kidney section. Glomerular tuft immunopositive area was quantified and data were reported as mean area of Col IV stain per glomerular tuft. Data were quality controlled by the study pathologist (BLH).

#### Quantitative analysis of mean PAS staining area per glomerular tuft

Whole-slide images of PAS stained sections were obtained with Aperio AT2 whole slide scanner. Glomerular tufts were manually selected in every other Quadrat for image analysis with Definiens Tissue Studio. An average of 41 tufts was selected per kidney section. PAS positive glomerular mesangial matrix area was quantified and the data were reported as mean area of mesangial PAS positive stain per glomerular tuft. Glomerular tuft area was quantified and the data were reported as mean area of glomerular tufts. Data were quality controlled by the study pathologist (BLH).

#### Semi-quantitative assessment of trichrome stained kidney

A board-certified veterinary pathologist (BLH) scored trichrome stained kidney sections in an iterative and blinded fashion. Initially, a portion of slides in the lean and obese groups were randomly selected and distributed in five piles based on the extent and distribution of trichrome stained glomerular capsule, glomerular tuft, and tubulointerstitial trichrome staining. The severity ranked from 1 (minimal evidence of fibrous expansion in the glomerular capsule, glomerular mesangium, or interstitium) to 5 (greatest severity of fibrous expansion). Next, 5 slides representative of severity scores 1 through 5 were selected. The remaining slides were returned with the study slides and scored blindly. If there was any uncertainty in assignment of an appropriate severity score, the slides were compared to the 5 severity score benchmark slides.

#### Staining for WT-1 and calculation of podocyte number per glomerulus

Immunohistochemical staining was performed on a Leica Bond RX® automated stainer (Leica Biosystems). The Bond Polymer Refine DAB Detection kit (Leica Biosystems, 9800 DAB) was used and all procedures followed manufacture’s preset protocols. The sections were subjected to ethylenediaminetetraacetic acid (EDTA) based antigen retrieval for 20 minutes followed by application of monoclonal rabbit anti-Wilms Tumor Protein antibody (Abcam, Cat#89901) at 0.3 μg/mL (1:800 dilution of a starting concentration of 0.247mg/ml) for 30 minutes at room temperature. An average of 44 glomeruli per animal were analysed for the number of WT-1^+^ cells per glomerular cross-sectional profile and glomerular tuft area. The Weibel-Gomez method was then used to determine the calculated number of podocytes per glomerulus (N_pod,glom_) [14, 15]. Briefly, the number of podocyte nuclear profiles per unit area of sectioned glomerulus (N_Apod,glom_) was determined by dividing the number WT-1^+^ cells per tuft by the tuft area. The nuclear areal fraction in a single section through the glomerulus (A_Apod,glom_) was determined by dividing the average tuft area by the average WT-1 staining area per tuft. The number of podocyte nuclei per unit glomerular volume (N_Vpod,glom_) was calculated using the equation N_Vpod,glom_ = (*K/*β) ∙ SQRT [(N_Apod,glom_)^3^/(A_Apod,glom_)], using a distribution coefficient (*K*) of 1.1 and a nuclear shape coefficient (β) of 1.45. An estimate of glomerular volume (V_glom_) was determined by the Weibel-Gomez method using the equation V_glom_ = average tuft area^1.5^ ∙ β/*K*, using a shape coefficient (β) of 1.38 for a sphere and a distribution coefficient (*K*) of 1.01. The estimated total number of podocytes per glomerulus was then calculated using the equation N_pod,glom_ = N_Vpod,glom_ ∙ V_glom_.

### ELISA for urinary Collagen type III breakdown product (uC3M)

The competitive ELISA for uC3M against the MMP-9 generated neo-epitope KNGETGPQGP was run as described previously [16–18]. Briefly, a 96-well plate pre-coated with streptavidin was further coated with 100 μL of 2.5 ng/mL synthetic peptide KNGETGPQGP-K-biotin dissolved in 10 mM PBS-BTB pH 7.4 buffer at 20°C for 30 minutes by constant shaking at 300 rpm. The plate was then washed five times in washing buffer. 20 μL of sample was then added, followed by 100 μL of 18 ng/mL peroxidase-conjugated anti-human mAb NB51–134 in 400 mM Tris–BTB pH 8.0. The free peptide, KNGETGPQGP, was used to generate a standard curve at 100, 50, 25, 12.5, 6.25, 3.12, 1.56 and 0 ng/mL concentrations. The plate was incubated for 20 hours at 4°C with constant shaking at 300 rpm. The plate was again washed five times. Finally, 100 μL tetramethylbenzinidine (TMB, Kem-En-Tec, Cat# 438OH) was dispensed and the plate was incubated in the dark for 15 minutes with shaking at 300 rpm. 100 μL of stop solution (0.1% H_2_SO_4_, Merck, Cat# 100731) was added and the plate was analysed in an ELISA reader at 450 nm with 650 nm as the reference.

### Transmission electron microscopy (TEM)

Kidney samples (n = 27 total) from all lean and obese rats at 29 weeks (5 lean, 5 obese), 34 weeks (5 lean, 5 obese), and 41 weeks (4 lean, 3 obese) were collected at necropsy and fixed by immersion in 4% formaldehyde/1% glutaraldehyde. All samples were trimmed and held at 4°C until processed for TEM. For processing, samples were rinsed three times for 10 minutes in 0.1 M phosphate buffer, followed by post fixation in 1% phosphate buffered osmium tetroxide for 2 hours at 4°C. Following osmication, samples were rinsed in deionized water followed by dehydration through a graded alcohol series. Samples were then transferred to resin, infiltrated for at least 2 hours under vacuum, and cured at 60°C for at least 24 hours prior to sectioning. Samples from the first three rats in each group (N = 18 total) were thick sectioned at 0.5 microns, trimmed to contain representative glomeruli, thin sectioned at approximately 90 nm, and stained with uranyl acetate and lead citrate. The first two samples in each group (N = 12 total) were viewed by a board-certified veterinary pathologist (TPB) in a Hitachi H-7100 transmission electron microscope.

### RNA sequencing of glomerular enriched tissue

Glomerular enriched tissue was isolated by mechanical sieving of kidney cortex using an established protocol [19]. Briefly, fresh cortex tissue (approximately one and a half kidneys per animal) was passed through a serial stack of mesh filters of 180, 106, and 75μ pore size. An optimal yield and enrichment of glomeruli was obtained by gently forcing cortex tissue through the 180μ mesh with the flat end of a 30 mL syringe plunger, followed by gravity flow collection on the lower mesh sizes with large volume PBS rinses. Materials trapped on the 106μ and 75μ mesh filters were collected in PBS by gentle flushing towards and aspiration from the edge of the mesh filter, combined, and placed back on a clean 75μ mesh for additional large volume PBS rinses and final collection. The material was then pelleted, resuspended in Qiazol (Qiagen Cat# 79306), and stored at -80°C prior to RNA isolation. Total RNA was isolated for all samples after study termination on the same day using miRNeasy RNA isolation kits (Qiagen Cat# 217004) on a Qiacube instrument (Qiagen). For all samples, RNA quality and integrity was confirmed on an Agilent 2100 bioanalyzer. RNA sequencing was performed on oligo(dT) purified poly(A)^+^ mRNA. A standard TruSeq mRNA library was constructed using TruSeq RNA Sample Prep Kit v2 (Illumina, Cat.# RS-122-2001). The library was sequenced by Illumina HiSeq 2000 using a paired-end run (2×100 bases). After sequencing, 40M 100 bp pair-end reads were generated for downstream analysis.

### RNA sequencing data and pathway enrichment analyses

Reads were mapped to a merged rat whole genome sequence from Ensemble Release 80 (ftp://ftp.ensembl.org/pub/release-80/fasta/rattus_norvegicus/dna/). The rat gene annotation in GTF format was downloaded from Ensembl (ftp://ftp.ensembl.org/pub/release-80/gtf/rattus_norvegicus). Our group has found Ensembl annotation to be more comprehensive than other gene annotations [20, 21]. Uniquely mapped reads were counted using featureCounts [21] and a gene counts table was generated. For each age group, the obese group was compared with the lean group and genes with fold change greater than two, an average expression higher than 0.5 Reads Per Kilobase per Million reads (RPKM), and a Benjamini-Hochberg adjusted p-value < 0.05, were reported as Differentially Expressed Genes (DEGs). All the raw sequencing reads have been submitted to the NCBI Sequence Read Archive, and are available under accession number SRP094779. This dataset can be explored interactively through the following GitHub link: https://interactivereport.github.io/ZSF1. Pathway analysis was performed using QIAGEN’s Ingenuity Pathway Analysis (IPA). DEGs with FDR < 0.05 were populated into the IPA Knowledge Base. A p-value < 0.05 was set as the significance threshold for enriched pathways. A z-score algorithm was applied to determine if an enriched pathway was up- or down-regulated based on the input DEGs. Enriched pathways identified in this study were compared to a published dataset of micro-dissected human DN glomeruli (GSE30528) [22]. Raw mRNA expression data files (CEL files) were processed and normalized using Robust Multi-array Average [23]. Differentially regulated genes were identified using LIMMA (Linear Models for Microarray Analysis) [24, 25]. Log fold changes were calculated for each transcript, and a false discovery rate (FDR) of 0.05 was set as the threshold of statistical significance. DEGs with FDR < 0.05 were populated into the IPA Knowledge Base for comparison with enriched pathways in the current analysis.

### Statistical methods

For quantitative clinical chemistry and histology measurements, statistical analyses were performed for two comparisons: 1) obese versus lean within each age group (denoted by *); and 2) obese versus 12-week-old obese (denoted by †). A p-value of ≤0.05 as determined by a two-way ANOVA Tukey’s multiple comparisons test was considered significant.

## Results

Cohorts of ZSF1 lean and obese animals were sacrificed at 12, 20, 24, 29, 34 and 41 weeks of age for analysis. One lean and two obese animals died over the study duration; therefore, the study in its entirety consisted of five lean and five obese animals per age group with the exception of week 41 animals, which consisted of four lean and three obese animals. An overview of the study is provided in S1 Fig.

**Fig 1:**
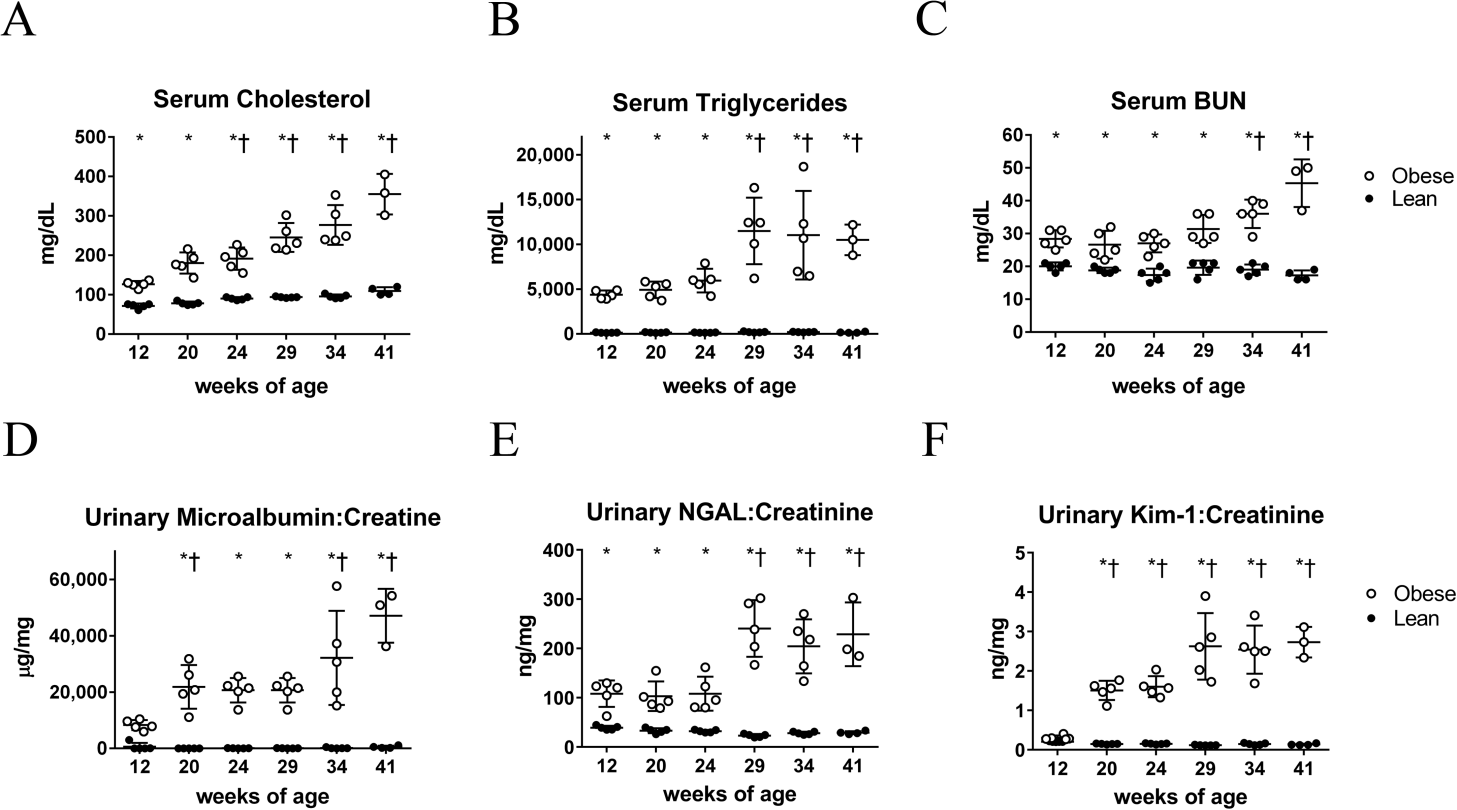
Obese ZSF1 rats have metabolic syndrome and evidence of renal impairment by 12 weeks of age. (A) Serum total cholesterol, (B) serum triglycerides, (C) serum blood urea nitrogen (BUN), (D) urinary microalbumin to creatinine ratio, (E) urinary NGAL to creatinine ratio, and (F) urinary Kim-1 to creatinine ratio in lean and obese animals over the study duration. Each point represents a single animal, with obese animals represented by open circles and lean animals represented by filled circles. Statistical analyses, here and in subsequent Figures, are for each obese group versus age-matched lean group (denoted by *) or for each obese group versus the 12-week-old obese group (denoted by †) using two-way ANOVA Tukey’s multiple comparisons tests.

Consistent with what is known about this model, obese ZSF1 rats displayed strong signatures of metabolic syndrome, renal injury, and progressive renal function decline. Serum cholesterol and serum triglycerides were elevated by 12 weeks of age, and increased as obese animals aged (Fig 1A and 1B). Serum blood urea nitrogen (BUN) values, a measure of renal function decline, were elevated by 12 weeks of age and further elevated at study termination (Fig 1C). Urinary microalbumin to creatinine ratios were elevated by 20 weeks of age and progressively increased over the study duration (Fig 1D). Indeed, at 41 weeks of age, the mean microalbumin to creatinine ratio was 90-fold higher in obese compared with lean animals (47,121 μg/mg versus 526 μg/mg). Consistent with renal injury, well characterized urinary markers of tubular injury, NGAL and Kim-1, were also elevated in obese animals ([26, 27]; Fig 1E and 1F), as was urinary N-acetyl glucosaminidase (NAG; S2 Fig). Other markers of metabolic syndrome, including reduced HDL cholesterol, elevated LDL cholesterol, elevated serum glucose, elevated serum non-esterified fatty acid (NEFA), and elevated serum insulin, were also evident in obese animals (S2 Fig). These results are consistent with previous reports of the presence of metabolic syndrome and early renal disease by 12 weeks of age, followed by progressively worsening renal function decline [8, 10–12, 28].

**Fig 2:**
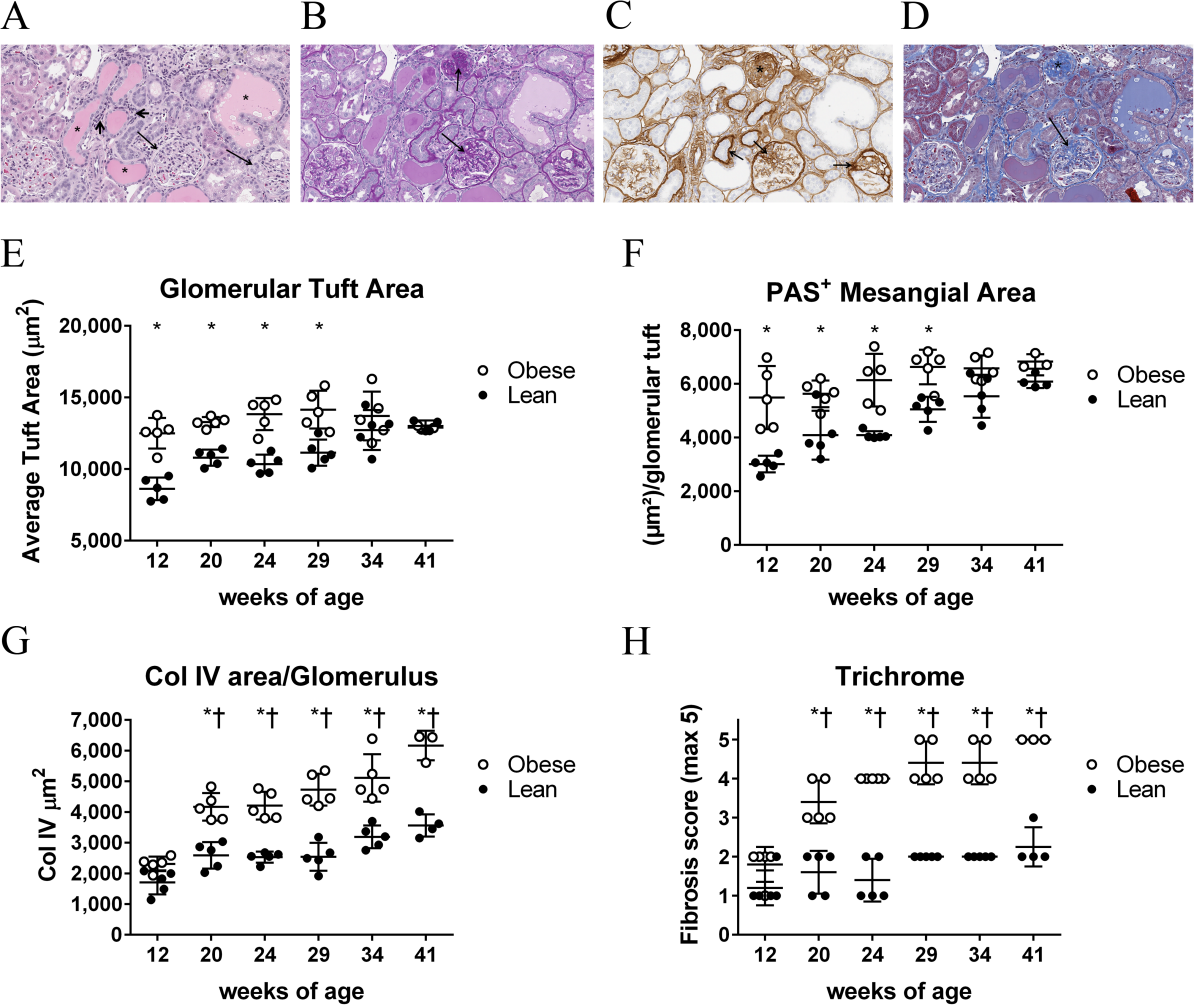
Obese ZSF1 have histologically evident renal fibrosis by 20 weeks of age that worsens over time. (A-D) Kidney section from a 34-week-old obese rat stained with (A) hematoxylin and eosin (H & E), (B) Periodic Acid Schiff (PAS), (C) Collagen IV (COL IV) IHC, or (D) trichrome. Highlighted features are as follows: (A) hyaline casts and proteinaceous material in dilated tubules (asterisk), thickened glomerular basement membranes (long arrows), and leukocytic infiltrates (short arrows); (B) thickened mesangial matrix (arrows); (C) increased deposition of collagen IV in glomerular mesangial matrix and basement membranes (asterisk and arrows); and (D) increased collagen in mesangial matrix (asterisk and arrows). (E) Glomerular tuft area as determined from H & E staining. (F) PAS positive glomerular mesangial area. (G) Results from Collagen IV immunohistochemistry expressed as Col IV staining area per glomerulus. (H) Results from blinded semi-quantitative trichrome staining for fibrosis severity.

We next performed quantitative and semi-quantitative histological analyses for fibrosis and structural alterations in the kidney. Representative stainings, showing a constellation of histological changes and severities in glomeruli, and representative tubular and interstitial leukocytic changes, are shown in Fig 2A–2D. The analyses revealed the following: a progression of glomerulosclerosis, increased thickness of Bowman’s capsule, and increased tubular casts and interstitial mononuclear leukocytic infiltrates in diseased animals (Figs 2A and S3). In 12-week-old obese rats, both glomerular tuft area (Fig 2E) and PAS^+^ glomerular mesangial matrix (Fig 2F) were elevated. However, there was no further increase in either parameter over the study duration, and the elevation observed at 12 weeks was obscured by an increase in tuft area and PAS^+^ mesangial matrix in lean rats as they aged. An increase in Collagen type IV staining area per glomerulus was evident in obese rats by 20 weeks of age, and there were significant differences in 12-week obese compared with other obese cohorts as well as in 12-week lean compared with 34- and 41-week lean (Fig 2G and data not shown). Similar trends were observed for Collagen type IV expressed as percent glomerular tuft area and as percent whole kidney (S4 Fig). A difference in overall trichrome staining in obese rats was evident by 20 weeks of age, and increased as obese rats aged (Fig 2H). The increase in Collagen type IV staining area, and overall fibrosis, was also reflected in increased levels of COL4A1, COL1A1, and COL3A1 mRNAs in whole kidney of obese animals (S5 Fig). Taken together, the histological findings demonstrate that glomerular hypertrophy and mesangial expansion are maximally manifested in obese animals by 12 weeks, and become obscured by an increase in these parameters in lean rats as they age. In contrast, renal fibrosis, as measured by Collagen type IV or trichrome staining, is persistently elevated in obese rats compared with lean rats, and increases in obese rats as they age.

**Fig 3:**
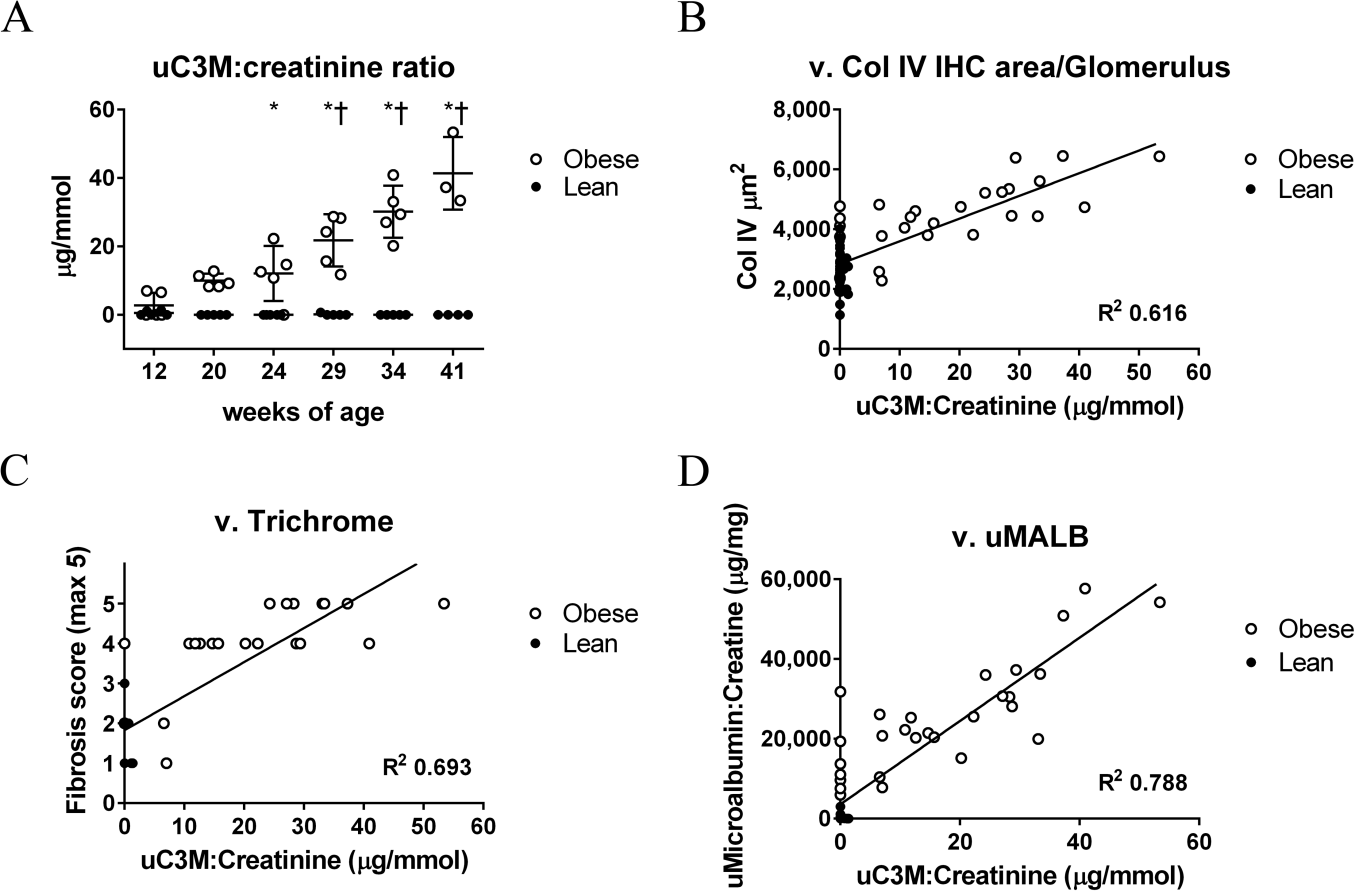
Levels of urinary Collagen type III breakdown product (uC3M) positively correlate with renal fibrosis and renal function decline in ZSF1 rats. (A) Levels of uC3M as determined by ELISA for the MMP9-generated neoepitope KNGETGPQGP, normalized to urine creatinine levels, in all animals over the study duration. Correlation of uC3M to creatinine levels versus Collagen type IV staining area per glomerulus (B), semi-quantitative trichrome staining (C), and microalbumin to creatinine ratios (D). R^2^ values, shown, were determined by linear regression of all values with obese animals represented by open circles and lean animals represented by filled circles.

Previously, the level of Collagen type III breakdown product in the urine (uC3M), measured by ELISA for the MMP-9 generated neo-epitope of Collagen type III, was described as a non-invasive biomarker of fibrosis in several models of renal fibrosis (5/6 Nx, anti-Thy 1.1, and adenine nephropathy models) [29]. We measured uC3M levels in ZSF1 rats longitudinally over the study duration. As shown in Fig 3A, uC3M to creatinine ratios were markedly higher in obese rats compared with lean rats from 24 weeks of age onwards, and increased over time to a maximum at study termination. Importantly, uC3M to creatinine ratios directly correlated with histological findings of fibrosis, including Collagen type IV staining area per glomerulus (Fig 3B; R^2^ 0.616) and semi-quantitative trichrome staining (Fig 3C; R^2^ 0.693). This is consistent with previous reports in other preclinical models linking uC3M levels to renal fibrosis. In addition, the uC3M to creatinine ratios directly correlated with microalbumin to creatinine ratios (Fig 3D; R^2^ 0.788). In ZSF1 rats, therefore, unlike in previously characterized models, uC3M appears to be a non-invasive biomarker not only for renal fibrosis but also for renal function decline (see Discussion).

**Fig 4:**
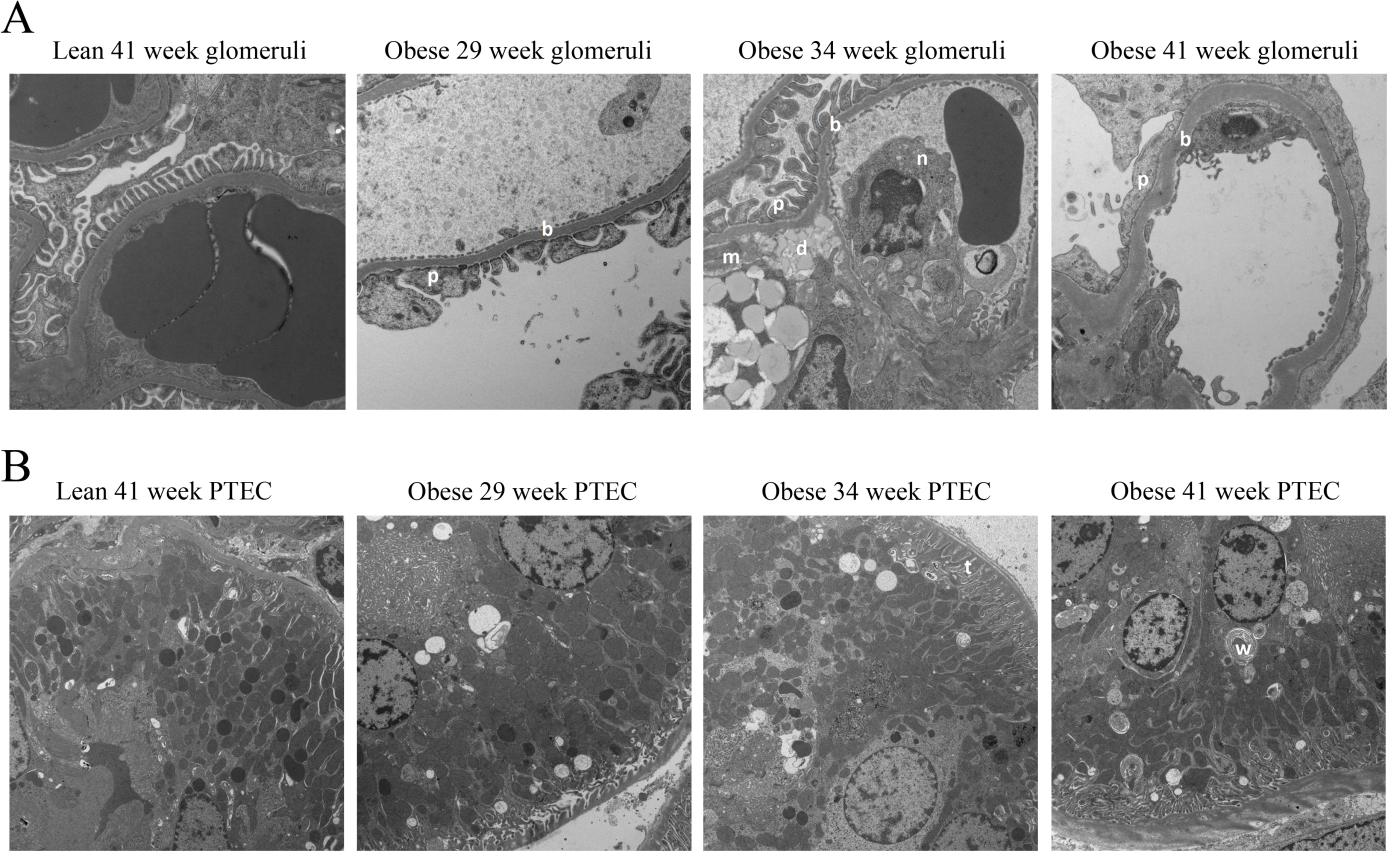
Nephropathy in obese ZSF1 rats is accompanied by ultrastructural changes in glomeruli and proximal tubular epithelial cells (PTEC). TEM of glomeruli (A) and PTEC (B) of a representative 41-week-old lean animal, and of representative 29-, 34-, and 41-week-old obese animals. Obesity and age-related findings were glomerular basement membrane thickening (b), podocyte foot process effacement (p), mesangial nodules (n) in glomerular capillary lumens, and increased mesangial matrix (m). Findings that were related to obesity, but not age, were glomerular lipid droplets (d) in mesangial cells, proximal tubular basement membrane thickening (t), and cytoplasmic membrane whorls (w). (Lead citrate/uranyl acetate, Magnification = 5,000x for A, and 2,000x for B).

To define ultrastructural changes associated with renal disease progression, we performed transmission electron microscopy of glomeruli and proximal tubular epithelial cells (PTEC) of selected animals. A representative week 41 lean animal and representative 29, 34, and 41 week obese animals were selected for depiction as described in Materials and Methods. No ultrastructural findings were present in the lean week 41 ZSF1 rat kidney. In contrast, ultrastructural findings were present in all obese groups in this analysis. Some of these findings were clearly also age-related in obese animals, while others were present indiscriminately in 29, 34, and 41 week obese animals. As indicated in Fig 4, findings that progressively worsened in obese animals with age were glomerular basement membrane thickening (minimal to moderate at 29 weeks, mild to moderate at 34 weeks, and moderate at 41 weeks); podocyte foot process effacement (minimal to moderate at 29 weeks, and moderate at 34 and 41 weeks); mesangial nodules composed of mesangial cells and matrix extending into capillary lumens (minimal to mild at 29 weeks, moderate at 34 weeks, and moderate at 41 weeks); and increased mesangial matrix (not present at 29 weeks, minimal at 34 weeks, and mild to moderate at 41 weeks). Ultrastructural findings that were present indistinguishably in week 29, 34, and 41 obese animals were proximal tubular basement membrane thickening (minimal to mild); whorls of membrane in proximal convoluted tubule cell cytoplasm (mild); and membrane bound lipid droplets in the cytoplasm of scattered mesangial cells (mild to moderate). No electron dense deposits suggestive of immune complexes were seen in subendothelial, intramembranous, or subepithelial glomerular capillary loop locations.

Glomerular injury and dysfunction, and in particular molecular and morphological changes in podocytes, are believed to contribute to the progression of diabetic kidney disease [30, 31]. To investigate gene expression changes in glomeruli over the course of disease progression, we performed global transcriptome profiling of glomerular-enriched tissue from all animals enrolled in the study. Tissue was obtained using an established method of mechanical sieving of freshly isolated kidney cortex through a series of sieves of decreasing pore size [19]. By light microscopy, preparations obtained in this way were predominantly glomeruli (>90%); however, as seen in S6 Fig, some tubules and tubulointerstitial cells were consistently present in these preparations. We therefore refer to these isolates as glomerular-enriched tissue as opposed to purified glomeruli. Poly(A)^+^ RNA from glomerular-enriched tissue was analyzed by Illumina RNA sequencing.

**Fig 5:**
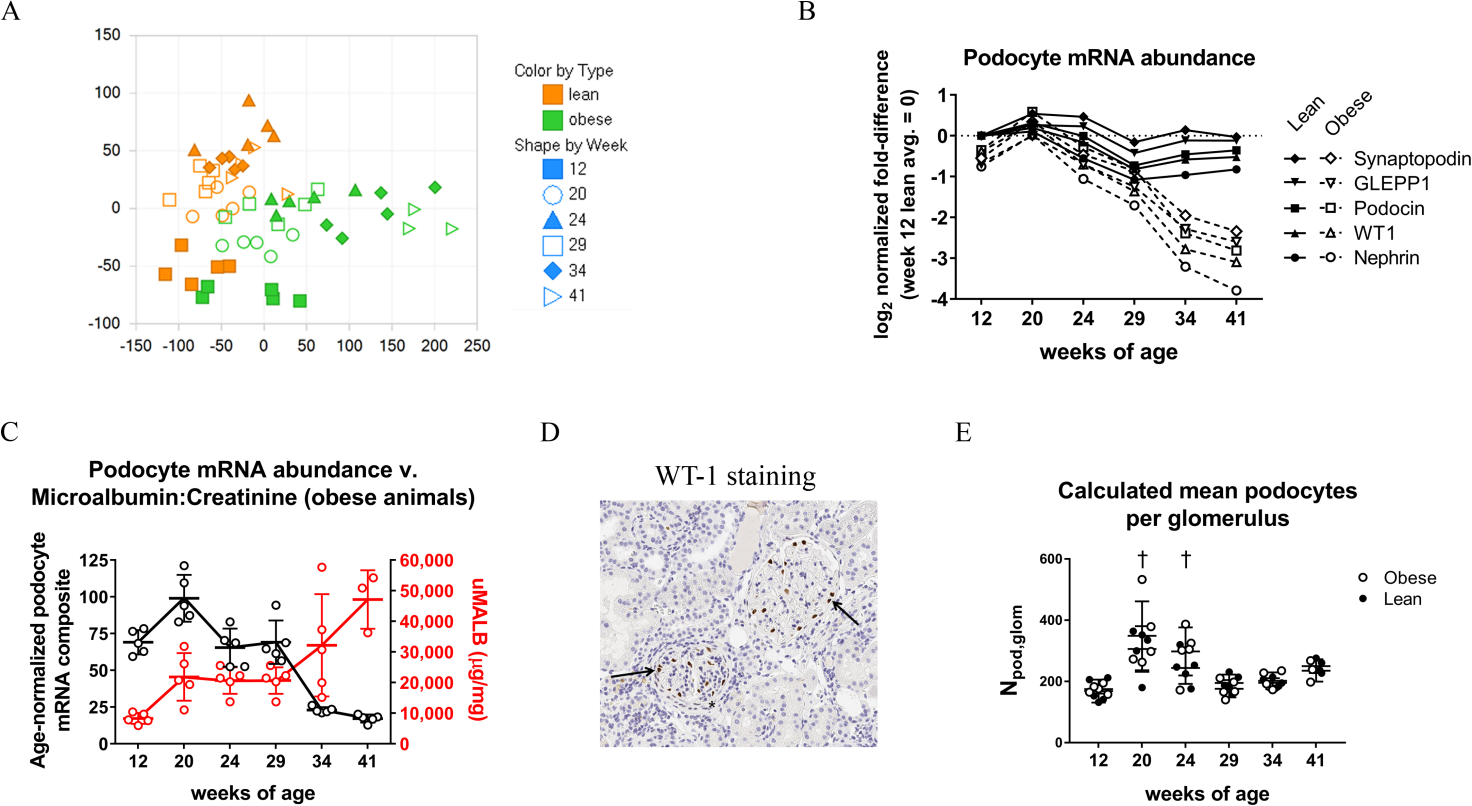
RNA Sequencing of glomerular-enriched tissue reveals disease- and age-related changes in both lean and obese ZSF1 rats. (A) Principal Component Analysis (PCA) of the union of all detectably expressed genes (average RPKM > 0.5) from RNA sequencing of poly(A)^+^ mRNA from glomerular-enriched tissue. (B) Analysis of selected podocyte-specific mRNAs in glomerular-enriched tissue. Each point represents the geometric mean of each cohort normalized to the geometric mean of 12-week-old lean animals (i.e., fold-change relative to 12-week-old lean). Data are plotted log2 normalized. (C) Podoycte mRNA “composite” (filled circles; left y-axis) and microalbumin to creatinine ratio (open circles; right y-axis) over the study duration. Composite podocyte mRNA levels are the average reduction in the five podocyte-specific genes shown in (B) normalized to age-matched lean animals (no reduction = 100%). (D) WT-1 staining from a representative 34-week-old obese rat. WT-1^+^ podocytes in two adjacent glomeruli are marked by arrows. One glomerulus has a thickened capsule (asterisk), and the other glomerulus is enlarged. (E) Calculated number of mean podocytes per glomerulus (N_pod,glom_) for all animals as determined by the Weibel-Gomez method (see Materials and Methods for details).

On average, 86% of raw sequenced reads were uniquely mapped to the rat reference genome, and 83% of uniquely mapped reads were in genomic exon regions. The number of differentially expressed genes between obese and age-matched lean animals (DEGs, as defined in Materials and Methods) increased in number over time from a week 12 value of 343 to a week 41 value of 2,134 (S7 Fig). Principal Component Analysis (PCA) of all detectable genes revealed a slight but discernible separation between the transcriptomes of glomerular-enriched tissues of lean and obese animals as early as 12 weeks of age (Fig 5A). In addition, there were age-related drifts in the transcriptomes of both lean and obese animals, with the largest separation between age-matched lean and obese animals occurring at the last time-point (week 41). The age-related drift in lean animals was noteworthy, as it has been speculated that changes in glomerular gene expression and morphology with age could contribute to the age-related incidence of ESRD ([32, 33]; S9 Fig and Discussion). Overall, the PCA analysis is consistent with a molecular signal of early renal disease by 12 weeks of age, age-related changes in the transcriptomes of both lean and obese animals over time, and of an increasingly divergent transcriptional profile between lean and obese animals with progressively worsening disease.

To interrogate these data for possible mechanistic insights, we examined the levels of podocyte-specific mRNAs in these glomerular-enriched tissues [34]. The mRNA levels of Synaptopodin, GLEPP1, Podocin, WT1, and Nephrin mRNAs for each cohort, normalized to the average expression levels in 12-week-old lean rats, are shown in Fig 5B. Podocyte-specific mRNAs clearly declined in obese animals with age, and this decline mirrored the increase in urinary microalbumin to creatinine ratio with an inflection point after the 29 week time-point (Fig 5C). To determine if this decline was due to a reduction in podocyte cell numbers, we performed immunohistochemistry using an antibody against Wilm’s Tumor 1 (WT-1) that stains the nuclei of podocytes [35–38]. A representative WT-1 staining image is provided in Fig 5D. The number of WT-1^+^ cells per glomerular cross-sectional, together with a calculated estimate of glomerular volume, was used to determine a calculated mean podocyte number per glomerulus (N_pod,glom_; [14, 15]). This analysis revealed an increase in N_pod,glom_ in both lean and obese animals at 20 and 24 weeks-of-age; however, there was no difference in N_pod,glom_ between lean and obese animals in any age group over the entire study duration (Fig 5E). By WT-1 staining, therefore, obese animals were indistinguishable from age-matched lean animals, a conclusion that was largely unchanged when these data were analyzed in different ways (number of WT-1^+^ cells per glomerular cross-sectional profile, calculated podocyte density per glomerulus, or podocyte mean nuclear size; S8 Fig). Taken together, the observed decrease in podocyte-specific mRNAs in the RNA sequencing analysis appears to be due to a reduced abundance of these mRNAs on a per-podocyte basis, and not to an overall loss in podocyte cell numbers in obese animals as they age.

**Fig 6:**
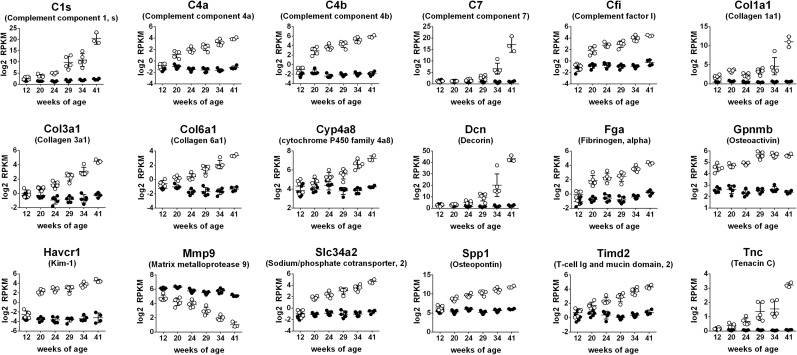
Longitudinal expression profiles of top p-value ranked transcripts in glomerular-enriched tissue from a comparison of 41-week-old animals. The top 18 genes from this analysis are presented in alphabetical order. Obese animals are represented by open circles, and lean animals by closed circles. Data are plotted as log2 RPKM reads per kilobase per million reads (RPKM).

By example, the longitudinal gene expression profiles of the top eighteen p-value-ranked DEGs at study termination (week 41) are provided in Fig 6. Present among the top genes identified in this way are complement factors (C1s, C4a, C4b, C7, Cfi), extracellular matrix and associated factors (Col1a1, Col3a1, Col6a1, Decorin, Tenacin C, Fibrinogen alpha, Osteoactivin, Osteopontin), an established biomarker of proximal tubule injury Kim-1 (also called Havcr1/Timd1), the related (in rodent) Timd2, the cytochrome P450 family member Cyp4a8, the sodium phosphate co-transporter SLC34a2, and the matrix metalloprotease MMP9, whose mRNA expression, unlike the others, decreased in obese rats over time. These data can be analysed and ranked in other ways, for example for gene expression changes that manifest in early disease, or that track with disease progression (S1 and S2 Tables, respectively). The annotated full dataset of all detectable genes from our study is provided in the NCBI Sequence Read Archive under accession number SRP094779.

**Fig 7:**
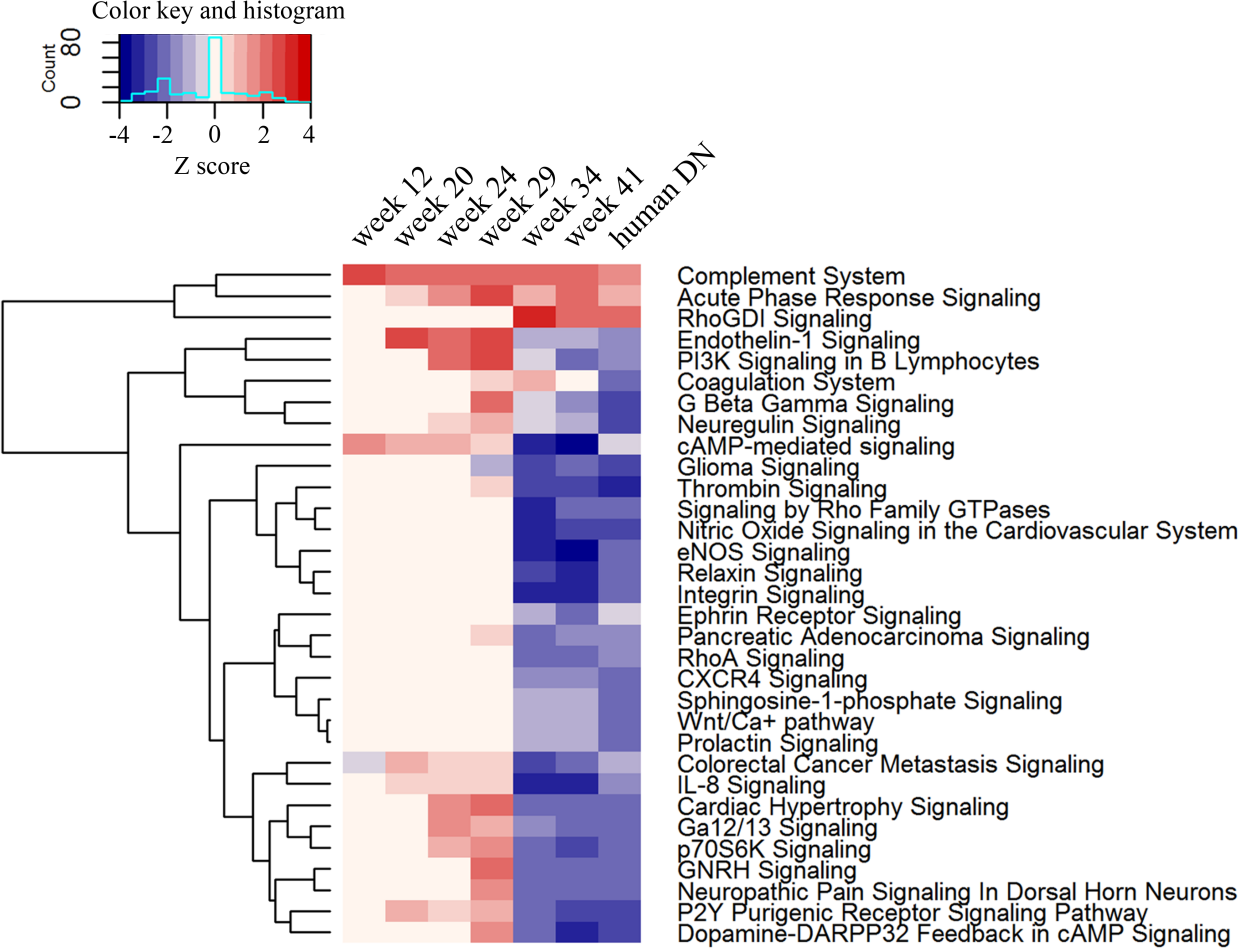
Pathway analysis reveals good concordance between weeks 34 and 41 obese ZSF1 animals and a published dataset of human DN glomeruli. Gene expression changes in glomerular-enriched tissues from obese ZSF1 rats at 12, 19, 24, 29, 34, and 41 weeks of age were from a comparison with age-matched littermates. Differentially expressed genes (DEGs) were populated in IPA Knowledge Base for pathway enrichment analysis. Gene expression changes in human type 2 DN were from Hodgin *et al*, and are of micro-dissected glomeruli analyzed by Affymetrix microarray [39]. A p-value was calculated using a right-tailed Fisher’s exact test to determine statistically significant over-representation of genes in any known canonical pathway. Activation Z scores indicate activation and inhibition of the pathways with |2| as threshold for significance. Numbers colored in red indicate pathway activation, while those in blue indicate pathway inhibition.

In order to gain insights in the translational relevance of this model to human DN, we compared the top DEG-enriched pathways to those from a published dataset of Affymetrix gene expression changes in micro-dissected human DN glomeruli (GSE30528) [22]. The comparison revealed a clear switch in glomerular gene expression between 29-week-old and 34-week-old ZSF1 obese rats (Fig 7). As a result, a good concordance with pathways affected in human DN emerges at the 34-week time-point, indicating molecular similarity in glomerular gene expression changes during disease in ZSF1 rats and in humans. This switch in global gene expression is coincident with the marked loss of podocyte-specific mRNAs, and the rise in urinary microalbumin to creatinine ratio, that is observed in obese animals between 29 and 34 weeks of age (Fig 5C).

**Fig 8:**
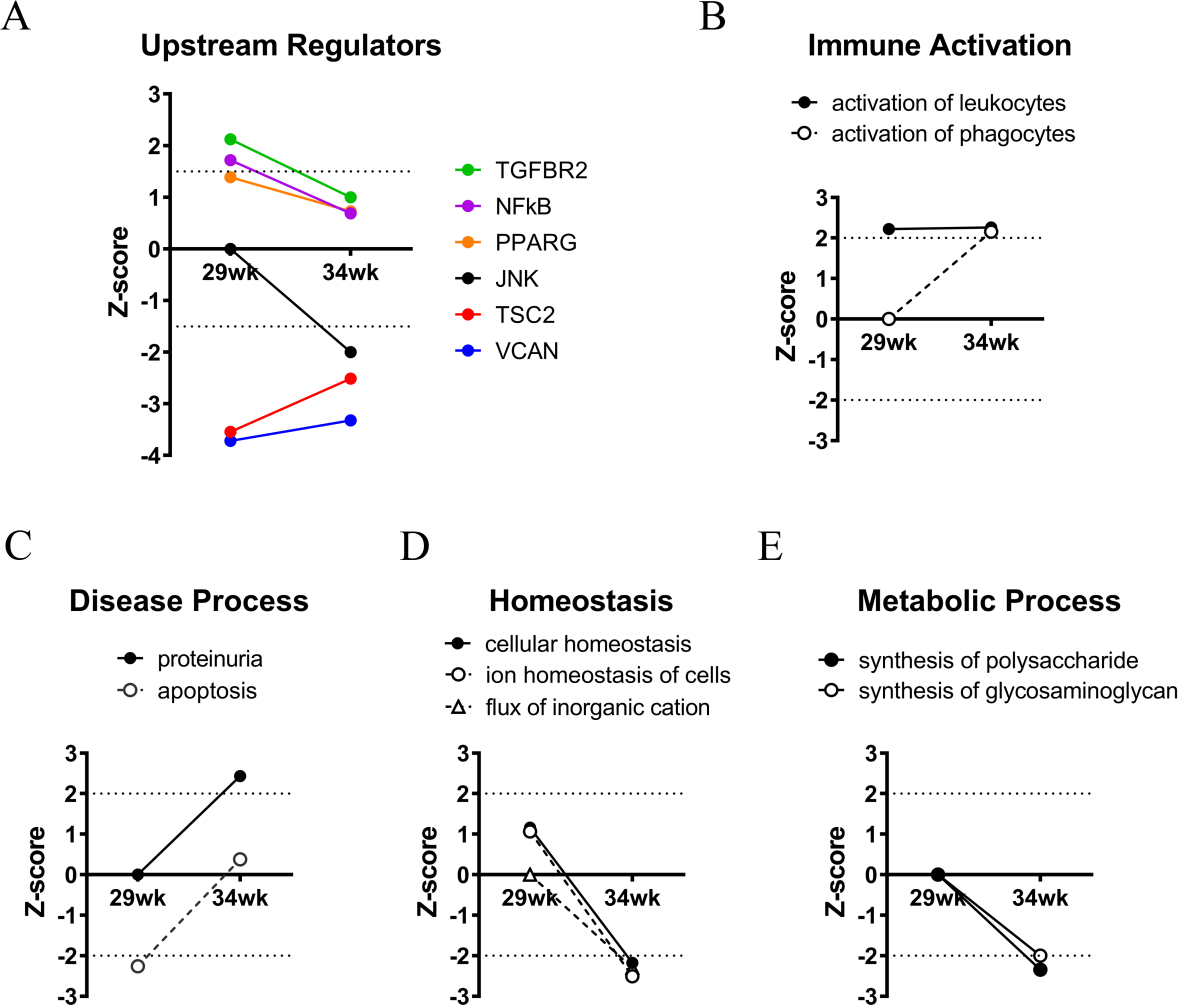
Pathway analysis of week 29 and week 34 animals reveals a switch in upstream regulators and cellular processes in ZSF1 obese animals. Differentially expressed genes (DEGs) for week 29 and 34 animals were populated in IPA, and pathway analysis was performed. (A) Upstream regulator analysis. (B-E) Diseases and functions analyses. Annotations related to relevant cellular and disease processes with significant Z-scores are represented. Z-scores are marked by a dotted line and indicate activation and inhibition of all pathways with Z-score ≥ |1.5| (A) or |2| (B-E) as threshold for significance.

We performed a comparative pathway analysis of DEGS from week 29 and week 34 obese animals to gain some understanding into biological processes associated with this transition (Fig 8). An analysis for all upstream regulators with Z-score ≥ |1.5| at either week 29 or 34 identified TGFBR2, NFκB, PPARG, JNK, TSC2, and VCAN (Fig 8A). While JNK was not identified in the 29 week dataset, it was sharply downregulated at 34 weeks. An analysis for downstream pathways with Z-score ≥ |2.0| revealed a signature of leukocyte activation at both time-points, and a signature of phagocyte activation only at week 34 (Fig 8B). In addition, signatures of proteinuria and apoptosis, two disease processes, increased between week 29 and week 34 (Fig 8C). Indeed, the apoptosis process appeared to be actively repressed at week 29, and this repression was lost at week 34. Finally, cellular processes related to homeostasis and metabolism were identified in the week 29 dataset but were significantly downregulated at week 34 (Fig 8D and 8E). Taken together, the downstream pathway analyses point to a potentially transformational event characterized by reduced homeostatic and metabolic function, failure to repress apoptosis, and activation of phagocytic cells in the glomeruli of ZSF1 obese animals as they transition to advanced DN.

## Discussion

Here we report a high resolution longitudinal analysis of progressive nephropathy in the obese ZSF1 rat, a model system that has gained interest as one that shares features with both early and late human DN. Our findings are consistent with previous reports that obese ZSF1 rats display metabolic syndrome and elevated microalbumin to creatinine ratio by 12 weeks of age, and progressively worsening renal fibrosis, inflammatory cell infiltration, and glomerulosclerosis that ultimately culminates in ESRD. This consistency is perhaps not surprising, given that ZSF1 rats are a spontaneous model of DN, however it does speak to the model’s reproducibility and robustness. Furthermore, cumulatively, ZSF1 rats appear to meet most if not all of the criteria put forth by the Animal Models of Diabetic Complications Consortium (AMDCC) for rodent models of progressive DN [40]. These criteria are: 1) greater than 50% decline in glomerular filtration rate (GFR) over the lifetime of the animal [8]; 2) greater than 10-fold increase in albuminuria compared with age- and gender-matched controls ([8, 10], and this study); and 3) pathology of kidneys showing mesangial matrix expansion, mesangiolysis, arteriolar hyalinosis, greater than 50% thickening of the glomerular basement membrane, and tubulointerstitial fibrosis ([8, 10, 12], and this study). While a decline in over 50% in GFR in this model has been reported by others, GFR decline is relatively modest in this model despite significant renal histopathology, and manifests only in older animals (e.g. in 44 week old animals but not in 24 or 32 week old animals) [8, 10, 28]. In previous work, it was proposed that hyperglycemia-induced hyperfiltration, in combination with higher protein intake due to hyperphagia, had an offsetting effect on GFR decline [10]. A limitation of the current study is that we did not measure GFR directly.

ZSF1 rats display a constellation of hemodynamic, metabolic, and renal complications commonly present in humans with DN, and several groups have tested pharmacological agents in this model accordingly. Bilan *et al*., showed that the PPAR-γ agonist Rosiglitazone, dosed from 8 to 32 weeks of age, reduced blood pressure, hyperglycemia, renal fibrosis, and albuminuria, in spite of pronounced drug-induced obesity (despite a marked reduction in food intake) [10]. PPAR-γ agonism therefore appears to elicit a metabolic reprogramming that is ultimately renoprotective in this model. In the same study, high dose angiotensin-converting enzyme (ACE) inhibitor Enalapril normalized blood pressure, prevented renal fibrosis, and virtually eliminated albuminuria. A supramaximal dose of Enalapril was used because several studies in humans have indicated that tissue protection by renin-angiotensin-aldosterone system (RAAS) inhibitors requires doses much higher than needed to normalize blood pressure [11, 41, 42]. Thus, clinical observations seen with Rosiglitazone and Enaplapril are largely recapitulated in obese ZSF1 rats. In addition, novel and emerging mechanisms have been tested in this model, with promising results [43–46]. In any pre-clinical study, it is important to know when to intervene in a manner appropriate to both the mechanism being tested and the disease stage when therapeutic dosing would likely begin in a clinical setting. Based on quantitative image analysis for PAS stained mesangial matrix and glomerular tuft area, Collagen IV area per glomerular tuft, and semi-quantitative trichrome assessment, our estimation is that the most relevant time to begin administering a molecule to assess for a direct and clinically translatable effect on nephropathy progression in obese ZSF1 rats would be between 24 to 29 weeks of age.

We provide evidence that the non-invasive biomarker urinary Collagen type III breakdown product, uC3M, increases over time in obese ZSF1 rats. uC3M was previously described as a marker of kidney fibrosis in the 5/6 Nx model, the Anti-Thy 1.1 induced nephropathy model, and the adenine nephropathy model [29]. In this earlier study, uC3M levels were markedly higher in all diseased animals compared with healthy controls. However, while uC3M levels correlated with the extent of renal fibrosis, they did not correlate with renal function decline as measured by proteinuria. In contrast, in this study, we find that uC3M levels correlate with both renal fibrosis and renal function decline in ZSF1 rats. In the previous study, tissue was analyzed at a single time-point not longitudinally as was done here; however, the reasons for this discrepancy are not entirely clear. None-the-less, taken together, the data suggest that MMP-driven collagen degradation, describing the dynamic process of tissue remodelling, may represent a non-invasive diagnostic approach to assess kidney fibrosis. uC3M levels have been examined in human chronic kidney disease, in this case in an IgA nephropathy (IgAN) cohort, and were found to inversely correlate with disease [16]. This could be due to different timeline between animals and humans, or due to different pathophysiology of IgAN and DN. Further studies will need to be performed to determine whether uC3M is a surrogate marker of fibrosis and renal function decline in human DN, as we report here for ZSF1 rats.

An understanding of the molecular changes accompanying renal disease progression to ESRD can be used to identify new diagnostic markers and therapeutic targets. In addition, molecular profiling provides information on how similar, or dissimilar, a particular model is to human disease. In an elegant study, Hodgin *et al*. used laser micro-dissection to analyze gene expression changes in glomeruli from three pre-clinical mouse models: a type I diabetes model (STZ-induced diabetes in DBA2 /J mice), an obese model of type 2 diabetes (db/db leptin receptor mutation on C57BLKS background; BKS db/db), and an obese and hypertensive model of type 2 diabetes (BKS db/db eNOS^-/-^ mice). Changes were then compared with those in glomeruli from a cohort of humans with type 2 DN [39]. Through this analysis, the authors identify glomerular transcriptional networks shared between preclinical disease models and humans but conclude that these animal models, at the time-points studied (22 weeks for DBA STZ, 24 weeks for BKS db/db, and 20 weeks for BKS eNOS^-/-^ db/db), most likely reflect changes in very early human disease. In contrast, and consistent with the progressive nature of nephropathy in ZSF1 rats, our longitudinal analysis revealed a strong concordance between week 34 onwards obese ZSF1 rats and the gene expression changes in human DN glomeruli reported by Hodgin *et al*. We note that putative tubule-specific genes, such as Kim-1 and Slc34a2, emerge from our analyses (Fig 6). This is due either to low level expression in glomeruli or, more likely, tubule contamination in our glomerular enriched tissue (S6 Fig). Based on our own analysis of the dataset from Hodgin *et al*., Kim-1 was detected and moreover, slightly upregulated, in micro-dissected human DN glomeruli.

One notable finding from these studies was that an increase in glomerular tuft area and PAS^+^ glomerular mesangial, observed in in 12-week-old obese animals, becomes obscured over time as both parameters increase in lean but not obese animals over time (Fig 2). In this regard, compared to 12-week-old lean animals, similar morphometric findings are observed in early disease animals (12-week-old obese animals) and in aged, non-diseased animals (41-week-old lean animals). In addition, by PCA analysis, there was a noticeable drift in the transcriptome of glomerular-enriched tissue from lean animals over time (Fig 5A). Indeed, a pathway analysis revealed that substantially more pathways are affected by age (lean 41-week-old animals versus lean 12-week-old animals) than in early disease (12-week-old obese animals versus 12-week-old lean animals; S9 Fig). This suggests that age itself is a major contributor to changes in glomerular gene expression and morphology, as has been previously reported in normotensive and non-diabetic rats [47, 48]. However, based on our analysis, an aged glomerulus does not resemble an early diseased glomerulus, at least at the transcript level, despite that they share some morphometric similarity. However, and as suggested by others, these underlying changes in the aged glomerulus could contribute to the age-related incidence of ESRD ([32, 33]

The RNA sequencing analysis of glomerular enriched tissue revealed a clear reduction in podocyte-specific mRNAs in obese animals as they aged. Compared to age-matched lean animals, 41-week old obese animals had a 74% reduction in Nephrin mRNA, a 79% reduction Podocin mRNA, 78% reduction in WT-1 mRNA, 76% reduction in Synaptopodin mRNA, and 78% reduction in GLEPP1 mRNA. A comparable decline in podocyte-specific mRNAs was present in the human DN glomeruli dataset from Hodgin *et al* (data not shown). This decline we observed was not, however, a result of an overall loss in podocyte cell numbers: as judged by WT-1 staining, the estimated number of glomerular podocytes was indistinguishable between age-matched lean and obese animals over the entire study duration (Fig 5E). The ZSF1 model may therefore differ in this regard from human DN, where it has been reported that disease and disease progression are associated with a loss of WT-1^+^ podocytes [35, 49, 50]. Our analysis cannot distinguish whether the WT-1^+^ cells are glomerular epithelial cells or of parietal epithelial origin. It does, however, suggest that there is a dramatic reduction in podocyte-specific mRNAs on a per-podocyte basis in diseased animals, in particular late in disease when there is robust proteinuria as well as ultrastructural evidence of podocyte effacement (Figs 4 and 5).

A concordance in glomerular gene expression changes between this model and the human dataset from Hodgin *et al* emerged in week 34 animals. A downstream pathway analysis revealed differences in cellular processes related to immune activation, apoptosis, cellular homeostasis, and metabolism between the week 29 time-point (when there was no concordance) and the week 34 time-point (when concordance emerged; Fig 8). In addition, several upstream regulators were identified in this analysis. TGFBR2, NFκB, and PPARG were potentially elevated regulators at both time-points. As mentioned, PPARγ agonists have been shown to be protective in this model, through an effect on systemic metabolism or, alternatively, a direct role in the renal compartment [51]. TSC2 and VCAN (Versican) were potentially repressed regulators at both time-points. The TSC2 gene product is a GTPase activating protein (GAP) that negatively regulates the mTORC1 complex [52], a master regulator of cellular metabolic reprogramming. Humans with loss-of-function mutations in TSC2 develop tuberous sclerosis complex (TSC), which, in addition to affecting multiple organ systems, has a number of renal manifestations [53]. While some differences in Z-scores between the 29 and 34 week time-points were evident for TGFBR2, NFκB, PPARG (upregulated at both 29 and 34 weeks), and TSC2 and VCAN (downregulated at both 29 and 34 weeks), these differences were relatively minor. However, a large difference was observed for the upstream regulator JNK, which was not identified in the week 29 dataset but was sharply depressed at week 34. The role of JNK in chronic kidney disease is unclear. In non-diabetic kidney disease models, JNK inhibitors reduced inflammation, fibrosis, cellular apoptosis, and renal function decline, indicating a disease-promoting role for JNK [54–57]. In contrast, in a model of type 1 diabetic kidney disease, inhibition of JNK led to a reduction in podocyte-specific mRNAs and a worsening of proteinuria, indicating a protective role [58]. Similarly, in the db/db mouse model of type 2 diabetes, inhibition of JNK resulted in increased podocyte damage and exacerbated albuminuria [59]. While the precise role of JNK in chronic kidney disease remains to be determined, our analysis identifies it as a key depressed regulator in obese ZSF1 rats when glomerular gene expression changes become concordant with that in biopsy-proven human DN.

In summary, our findings provide further evidence that the obese ZSF1 rat model represents one of the more translatable available pre-clinical models for progressive human DN. Based on histological findings, we suggest that the optimal age to initiate dosing in studies designed to slow or halt the progression of nephropathy is between 24 and 29 weeks. In addition, we show that levels of Collagen type III breakdown product in the urine correlate with renal fibrosis and functional decline in ZSF1 rats, warranting additional studies of human DN cohorts. Finally, we demonstrate similarity in glomerular gene expression changes between ZSF1 rats and a published dataset of human DN glomeruli, suggesting an overlap in pathobiological mechanisms between this pre-clinical model and human disease.

## Supporting information

S1 Table Genes with differential expression at all time-points, including in early disease (week 12). A subset of DEGs from glomerular enriched tissue that passed statistical significance at all 6 time points was identified. The list was further filtered using a more stringent fold change cut-off of 3. The analysis identified 17 genes in obese animals with an altered expression pattern that is evident by 12 weeks of age and is sustained over the study duration. Each cell represents the corresponding log_2_ Ratio (obese vs lean).
(DOCX)

S2 Table Genes with a differential expression pattern that tracks with disease severity. Shown are genes that satisfied two criteria: first, they were not necessarily differentially expressed at week 12, but were differentially expressed at all later time points; and second, their fold changes increased or decreased progressively over the study duration. None of these genes changed significantly over time in lean animals (data not shown). Each cell represents the corresponding log_2_ Ratio (obese vs lean).
(DOCX)

S1 Fig Study overview. Age and number of lean and obese ZSF1 animals analyzed per group, and summary of endpoints. At each time-point, blood and urine was collected and kidneys were prepared for the indicated analyses. The levels of a Collagen type III breakdown product in urine (uC3M) were measured by ELISA for the MMP9-generated neo-epitope KNGETGPQGP, as described in Methods. For renal histology, H & E, Col IV, PAS, and trichrome staining were performed. Transmission electron microscopy (TEM) was performed on a representative week 41 lean animal and on representative week 29, 34, and 41 obese animals. RNA sequencing of poly(A)^+^ mRNA from glomerular-enriched tissue was performed on all animals enrolled in the study.
(TIF)

S2 Fig Other metabolic and renal blood/urine parameters. Shown are serum cholesterol (LDL, HDL), serum glucose, serum non-esterified fatty acids (NEFA), and urinary N-Acetyl Glucosaminidase (NAG, a marker of tubular injury), serum creatinine, serum insulin, and serum glucose-to-insulin ratio. Serum creatinine levels, shown in the lower right panel, varied slightly between lean and obese animals over the study duration.
(TIF)

S3 Fig Immunohistochemistry (IHC) for immune cell infiltrates in a kidney section from a representative 34-week-old obese animal. Left, Iba-1 staining for monocytes and macrophages; Iba-1^+^ cells are present in glomeruli and the interstitium (arrows). Right, CD3 staining for T-cells. CD3^+^ cells are present in the interstitium (arrows).
(TIF)

S4 Fig Additional histological measurements to complement Fig 3. Results from Collagen IV immunohistochemistry expressed as percent Col IV staining area per glomerular tuft area (left), or percent Col IV staining area of whole kidney (right).
(TIF)

S5 Fig Taqman analysis of total RNA isolated from whole kidney tissue. A statistical difference in the expression of these fibrotic mRNAs between age-matched lean and obese animals is evident by 19 weeks of age (Col4a1 mRNA), 24 weeks of age (Col1a1 mRNA), and 29 weeks of age (Col3a1) mRNA. Reported 2^-dCt values are versus GAPDH mRNA.
(TIF)

S6 Fig Light microscopy image of a representative preparation of glomeruli isolated by mechanical sieving. Left, the preparation is predominantly glomeruli (*), however tubule (arrow) and tubulointerstitial cells are also present. Right, higher magnification image of glomeruli isolated in this manner. Scale bar 200 μM.
(TIF)

S7 Fig Overview of differentially expressed genes (DEGs) in glomerular-enriched tissue. (A) The number of DEGs from a comparison of age-matched lean and obese animals trends up over time. (B) Volcano plots for DEGs. The obese group was compared with lean group, and statistically significant (FDR ≤ 0.05) up-regulated and down-regulated genes with fold change greater than 2 are colored in red and blue, respectively.
(TIF)

S8 Fig Quantitation of WT-1+ staining to determine podocyte content within glomeruli. An average of 44 glomerli were analyzed for each animal. (A) Number of WT-1^+^ cells per glomerular cross-sectional area. (B) Mean glomerular volume (V_glom_) calculated from tuft area using the Weibel-Gomez method (see Materials and Methods). (C) Estimated podocyte density per glomerulus calculated by dividing the calculated number of podocytes per glomerulus (N_pod,glom_; Fig 5E and Materials and Methods) by V_glom_., expressed as podocytes/10^6^ cm^3^. (D) Mean podocyte nuclear size, expressed as WT-1 staining area per WT-1^+^ cell, in μm^2^.
(TIF)

S9 Fig Pathway analysis of age- and early disease-related gene expression changes in glomerular-enriched tissue. The increased glomerular tuft area (A) and PAS^+^ mesangial area (B) in 12-week old ZSF1 obese animals becomes obscured by an increase in both parameters in ZSF1 lean animals as they age (re-depiction of data provided in Fig 2). (C) Pathway analysis of “age-related” gene expression changes from a comparison of 41 week lean to 12 week lean animals. The bars represent the Z-score relative to the top x-axis, with activated and inactivated pathways in red and blue, respectively; the–Log(p-value) is depicted as a dot relative to the bottom x-axis scale. (D) Pathway analysis of “early disease-related” gene expression changes from a comparison of 12 week obese to 12 week lean animals. (E) Venn diagram of the 44 age-related and 4 early disease-related pathways. Only one pathway, OX40 signaling, is represented in both groups.
(TIF)
