# Supplementary material for: High resolution molecular and histological analysis of renal disease progression in ZSF1 fa/faCP rats, a model of type 2 diabetic nephropathy
Source: PLoS One. 2017 Jul 26;12(7):e0181861. doi: 10.1371/journal.pone.0181861 (PMC5529026; doi:10.1371/journal.pone.0181861)
Supplement: S1 Table — A subset of DEGs from glomerular enriched tissue that passed statistical significance at all 6 time points was identified. The list was further filtered using a more stringent fold change cut-off of 3. The analysis identified 17 genes in obese animals with an altered expression pattern that is evident by 12 weeks of age and is sustained over the study duration. Each cell represents the corresponding log2 Ratio (obese vs lean). (DOCX) [file pone.0181861.s001.docx]

**S1 Table**

| ***Genes with differential expression at all time-points, including in early disease (week 12)*** | | | | | | |
| --- | --- | --- | --- | --- | --- | --- |
| **Gene** | **12 weeks** | **20 weeks** | **24 weeks** | **29 weeks** | **34 weeks** | **41 weeks** |
| Angptl7 | 3.78 | 4.76 | 4.36 | 4.81 | 5.73 | 5.19 |
| Gnat2 | 3.13 | 4.04 | 3.39 | 3.41 | 1.63 | 1.61 |
| Trem2 | 2.86 | 3.66 | 3.87 | 4.25 | 4.42 | 4.65 |
| Siglec1 | 2.59 | 2.62 | 3.02 | 2.96 | 2.42 | 3.15 |
| Folr2 | 2.12 | 2.10 | 2.04 | 2.57 | 2.20 | 2.13 |
| Hmgcs2 | 2.09 | 2.25 | 2.06 | 2.05 | 2.16 | 1.71 |
| C2 | 2.07 | 2.00 | 2.49 | 2.77 | 2.51 | 3.21 |
| Hk3 | 2.04 | 1.66 | 2.37 | 2.82 | 2.21 | 2.76 |
| Mmp12 | 2.03 | 2.12 | 2.40 | 2.62 | 1.68 | 1.68 |
| Ncr2 | 2.02 | 2.16 | 2.25 | 2.31 | 1.71 | 1.89 |
| Gpx2 | 1.96 | 2.88 | 2.63 | 3.67 | 3.75 | 4.32 |
| Fabp4 | 1.89 | 2.99 | 3.45 | 3.62 | 3.52 | 2.58 |
| Col1a1 | 1.87 | 2.34 | 2.16 | 2.98 | 2.72 | 3.98 |
| Gpnmb | 1.86 | 2.08 | 2.54 | 3.11 | 2.63 | 3.03 |
| Fam184b | -1.91 | -1.69 | -1.67 | -1.70 | -2.30 | -2.92 |
| Olah | -2.44 | -2.44 | -2.52 | -1.72 | -2.62 | -2.66 |
| Prrt2 | -3.44 | -3.33 | -2.72 | -2.20 | -3.00 | -2.37 |
