# Supplementary material for: High resolution molecular and histological analysis of renal disease progression in ZSF1 fa/faCP rats, a model of type 2 diabetic nephropathy
Source: PLoS One. 2017 Jul 26;12(7):e0181861. doi: 10.1371/journal.pone.0181861 (PMC5529026; doi:10.1371/journal.pone.0181861)
Supplement: S2 Table — Shown are genes that satisfied two criteria: first, they were not necessarily differentially expressed at week 12, but were differentially expressed at all later time points; and second, their fold changes increased or decreased progressively over the study duration. None of these genes changed significantly over time in lean animals (data not shown). Each cell represents the corresponding log2 Ratio (obese vs lean). (DOCX) [file pone.0181861.s002.docx]

**S2 Table**

| ***Genes with a differential expression pattern that tracks with disease severity*** | | | | | | |
| --- | --- | --- | --- | --- | --- | --- |
| **Gene** | **12 weeks** | **20 weeks** | **24 weeks** | **29 weeks** | **34 weeks** | **41 weeks** |
| C4b | 0.88 | 4.51 | 5.96 | 6.42 | 6.78 | 7.63 |
| Havcr1 | 0.91 | 5.89 | 6.44 | 6.55 | 6.78 | 7.62 |
| Slc34a2 | 0.56 | 2.94 | 3.18 | 4.09 | 4.23 | 5.10 |
| C4a | 0.67 | 2.19 | 3.33 | 3.97 | 4.50 | 4.88 |
| Trem2 | 2.86 | 3.66 | 3.87 | 4.25 | 4.42 | 4.65 |
| Cfi | 0.45 | 2.76 | 3.43 | 3.94 | 4.53 | 4.53 |
| Col6a1 | 0.60 | 1.27 | 2.12 | 3.14 | 3.48 | 4.53 |
| Col3a1 | 0.19 | 1.01 | 2.12 | 3.15 | 3.47 | 4.53 |
| Fga | 0.90 | 2.67 | 2.85 | 3.26 | 3.57 | 3.92 |
| Timd2 | 0.51 | 1.22 | 1.96 | 2.81 | 3.03 | 3.36 |
| PCOLCE2 | 0.37 | 1.07 | 1.42 | 2.41 | 2.44 | 3.21 |
| Igfbp1 | 1.05 | 1.36 | 1.93 | 2.28 | 2.84 | 3.06 |
| Angptl4 | 1.51 | 2.30 | 2.40 | 2.64 | 2.64 | 2.92 |
| Hmox1 | 0.76 | 1.15 | 1.29 | 1.62 | 1.67 | 2.25 |
| Myc | 0.96 | 1.14 | 1.18 | 1.51 | 1.56 | 2.25 |
| Mab21l3 | 0.86 | 1.11 | 1.50 | 1.58 | 2.07 | 2.09 |
| Lyz2 | 0.78 | 1.13 | 1.31 | 1.57 | 1.59 | 1.96 |
| Mt2A | 1.06 | 1.07 | 1.14 | 1.35 | 1.71 | 1.90 |
| Kcnmb2 | -1.00 | -1.09 | -1.27 | -1.57 | -2.75 | -3.45 |
| Ccdc184 | -0.91 | -1.47 | -1.60 | -2.23 | -3.37 | -3.47 |
