# Supplementary figures and images for: High resolution molecular and histological analysis of renal disease progression in ZSF1 fa/faCP rats, a model of type 2 diabetic nephropathy
Source: PLoS One. 2017 Jul 26;12(7):e0181861. doi: 10.1371/journal.pone.0181861 (PMC5529026; doi:10.1371/journal.pone.0181861)

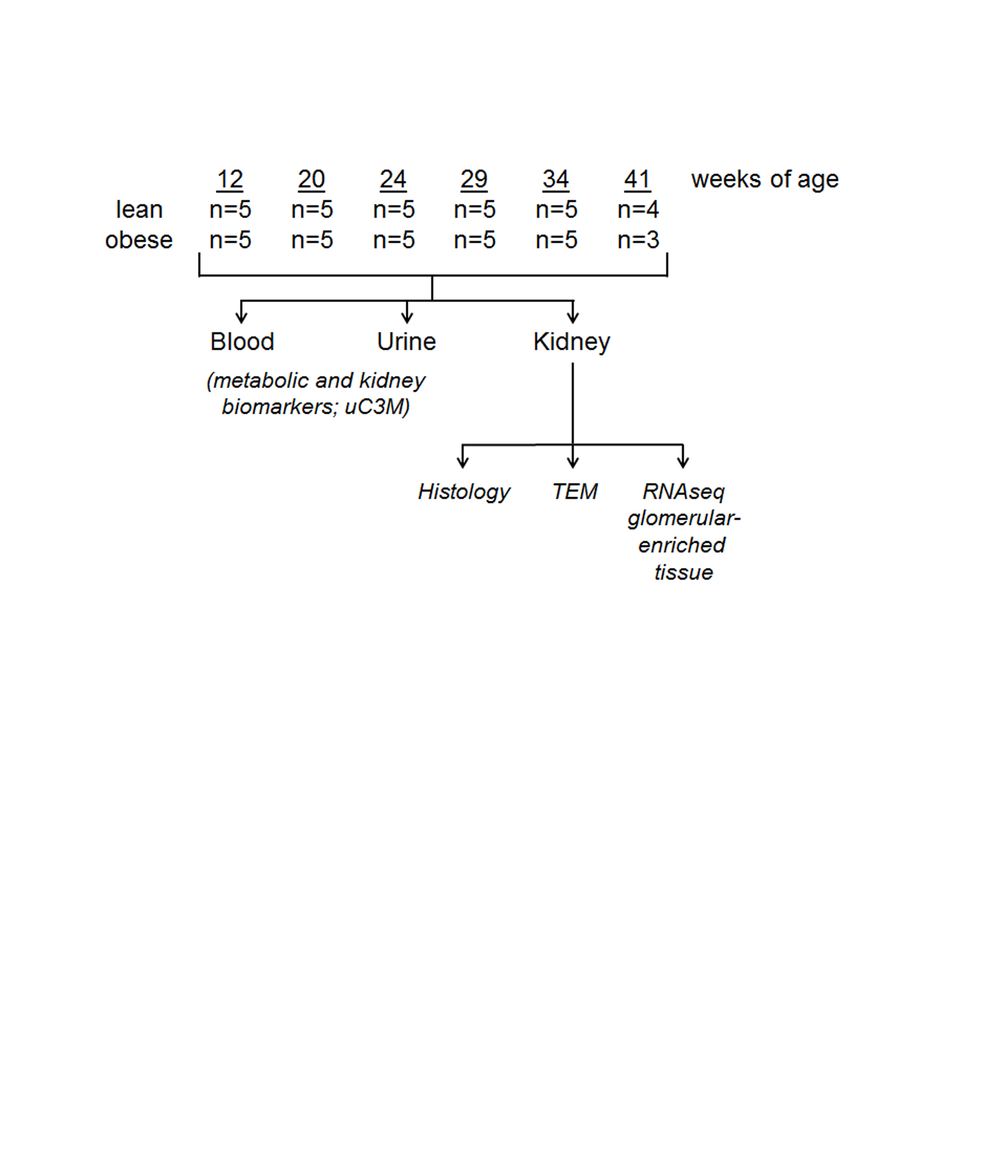

Supplement: S1 Fig — Age and number of lean and obese ZSF1 animals analyzed per group, and summary of endpoints. At each time-point, blood and urine was collected and kidneys were prepared for the indicated analyses. The levels of a Collagen type III breakdown product in urine (uC3M) were measured by ELISA for the MMP9-generated neo-epitope KNGETGPQGP, as described in Methods. For renal histology, H & E, Col IV, PAS, and trichrome staining were performed. Transmission electron microscopy (TEM) was performed on a representative week 41 lean animal and on representative week 29, 34, and 41 obese animals. RNA sequencing of poly(A)+ mRNA from glomerular-enriched tissue was performed on all animals enrolled in the study. (TIF) [file pone.0181861.s003.tif]

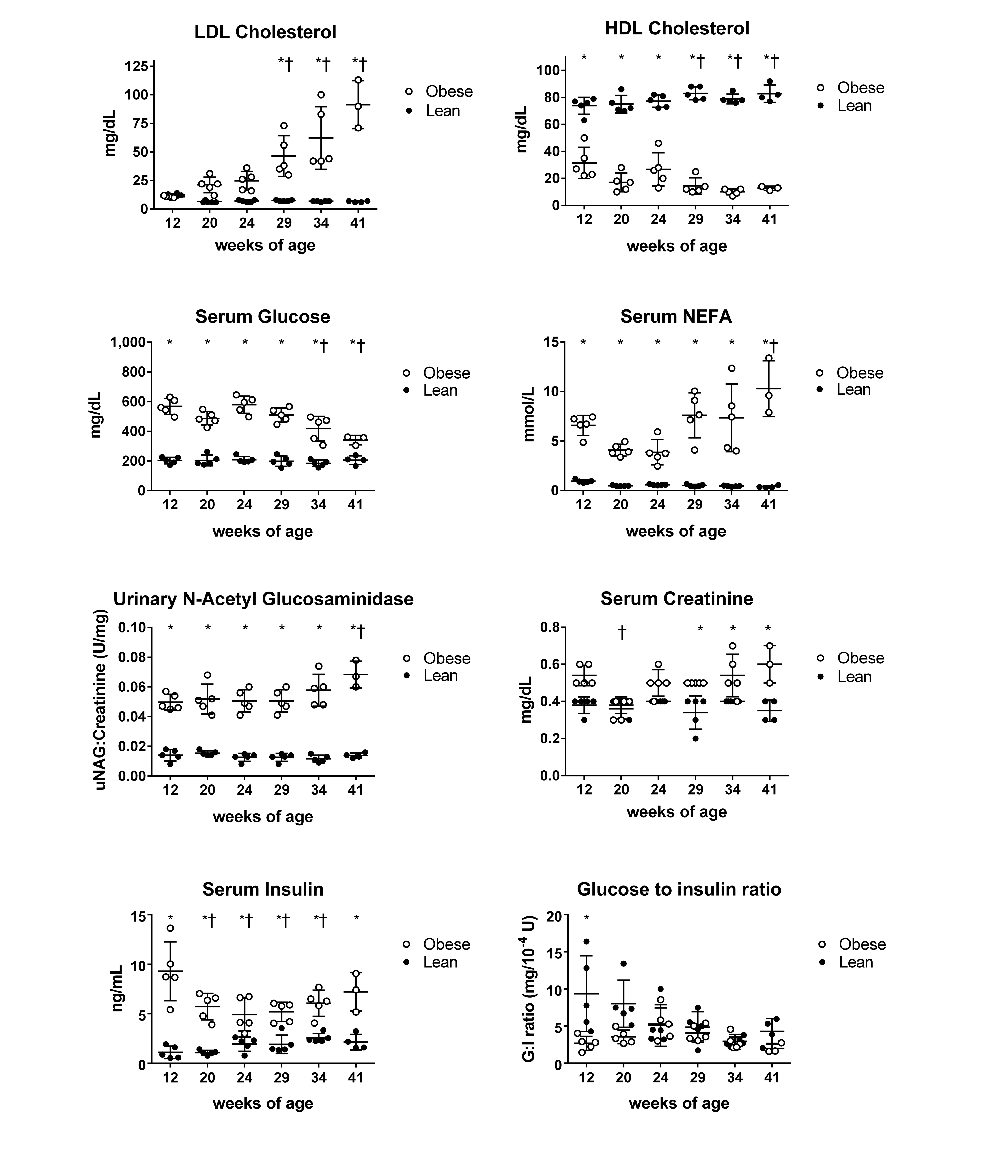

Supplement: S2 Fig — Shown are serum cholesterol (LDL, HDL), serum glucose, serum non-esterified fatty acids (NEFA), and urinary N-Acetyl Glucosaminidase (NAG, a marker of tubular injury), serum creatinine, serum insulin, and serum glucose-to-insulin ratio. Serum creatinine levels, shown in the lower right panel, varied slightly between lean and obese animals over the study duration. (TIF) [file pone.0181861.s004.tif]

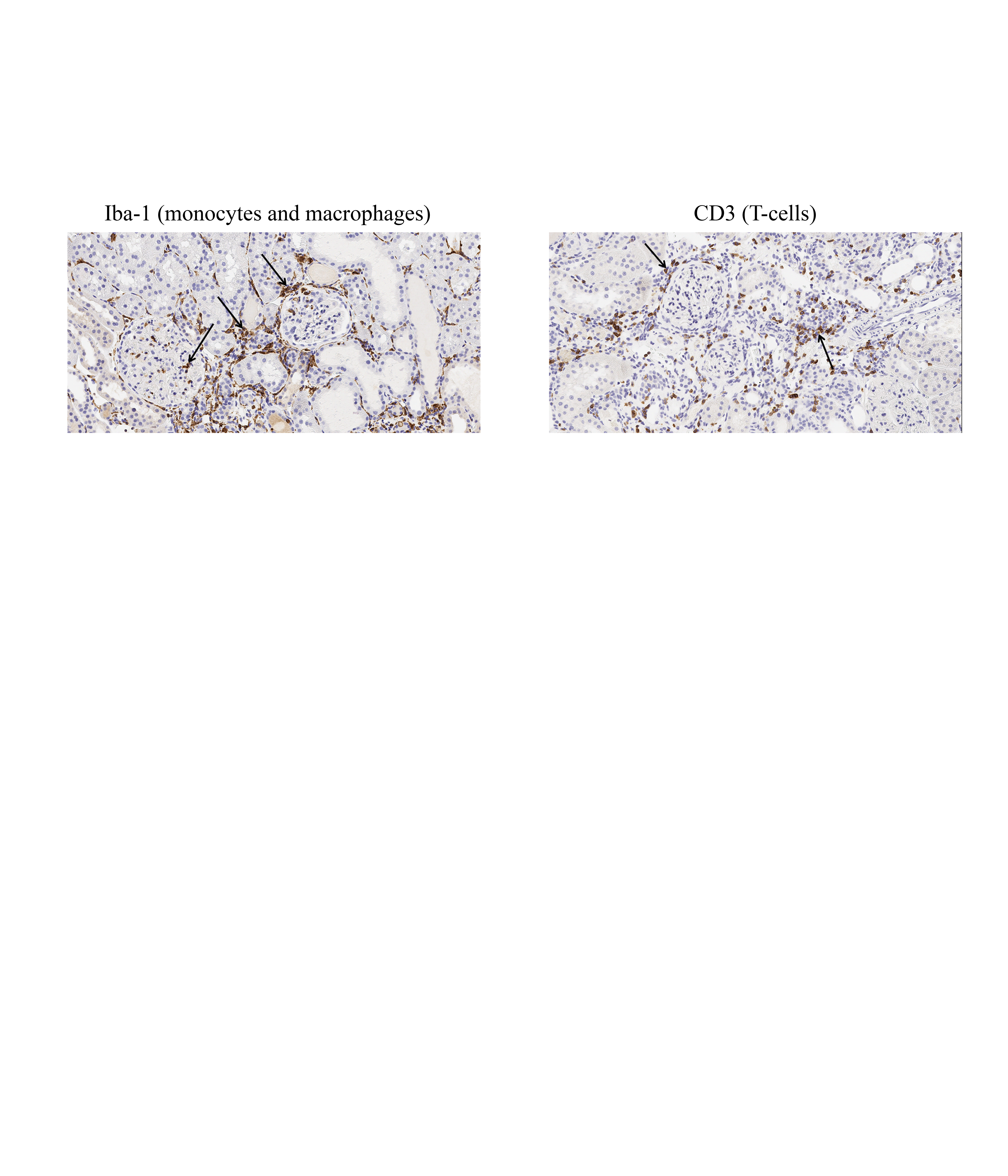

Supplement: S3 Fig — Left, Iba-1 staining for monocytes and macrophages; Iba-1+ cells are present in glomeruli and the interstitium (arrows). Right, CD3 staining for T-cells. CD3+ cells are present in the interstitium (arrows). (TIF) [file pone.0181861.s005.tif]

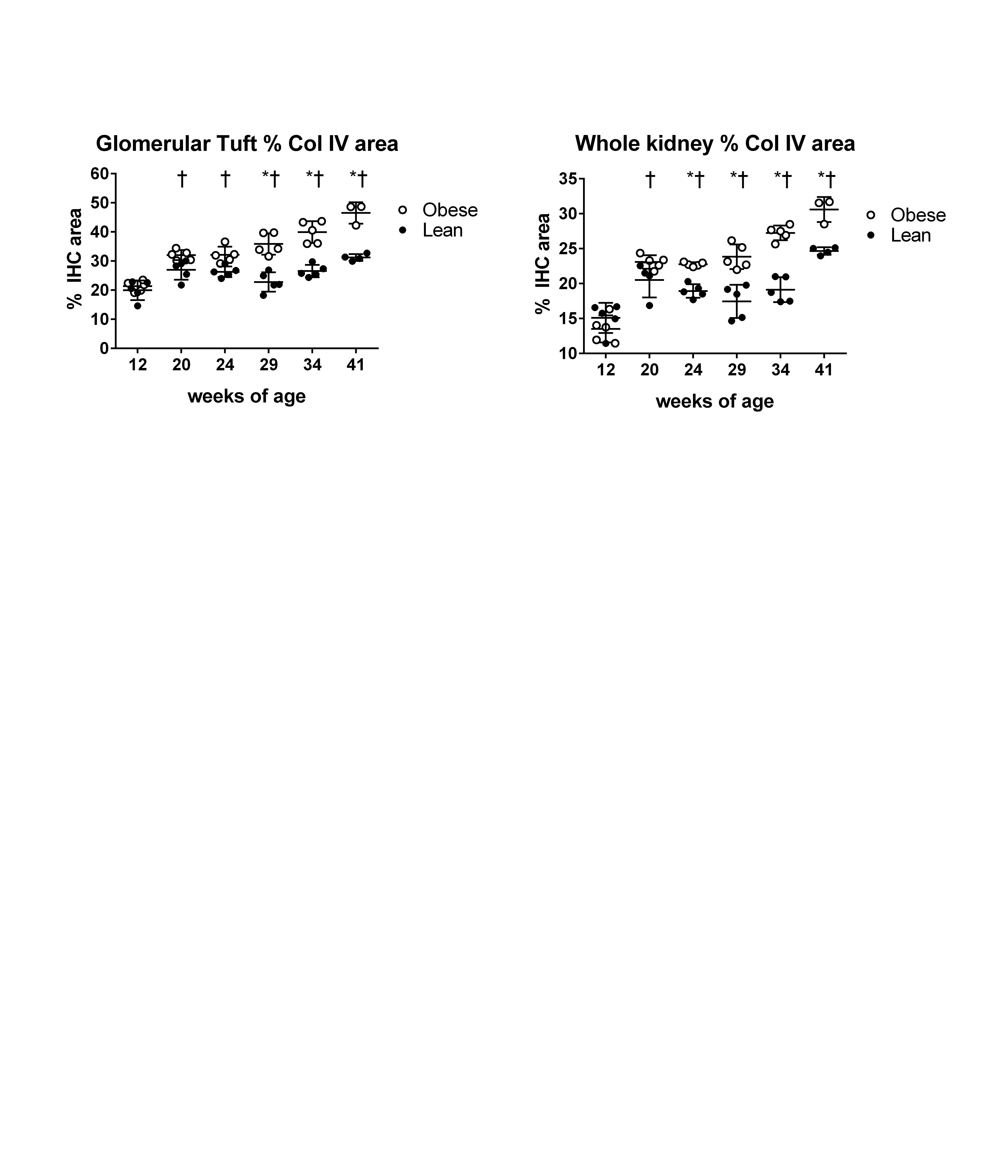

Supplement: S4 Fig — Results from Collagen IV immunohistochemistry expressed as percent Col IV staining area per glomerular tuft area (left), or percent Col IV staining area of whole kidney (right). (TIF) [file pone.0181861.s006.tif]

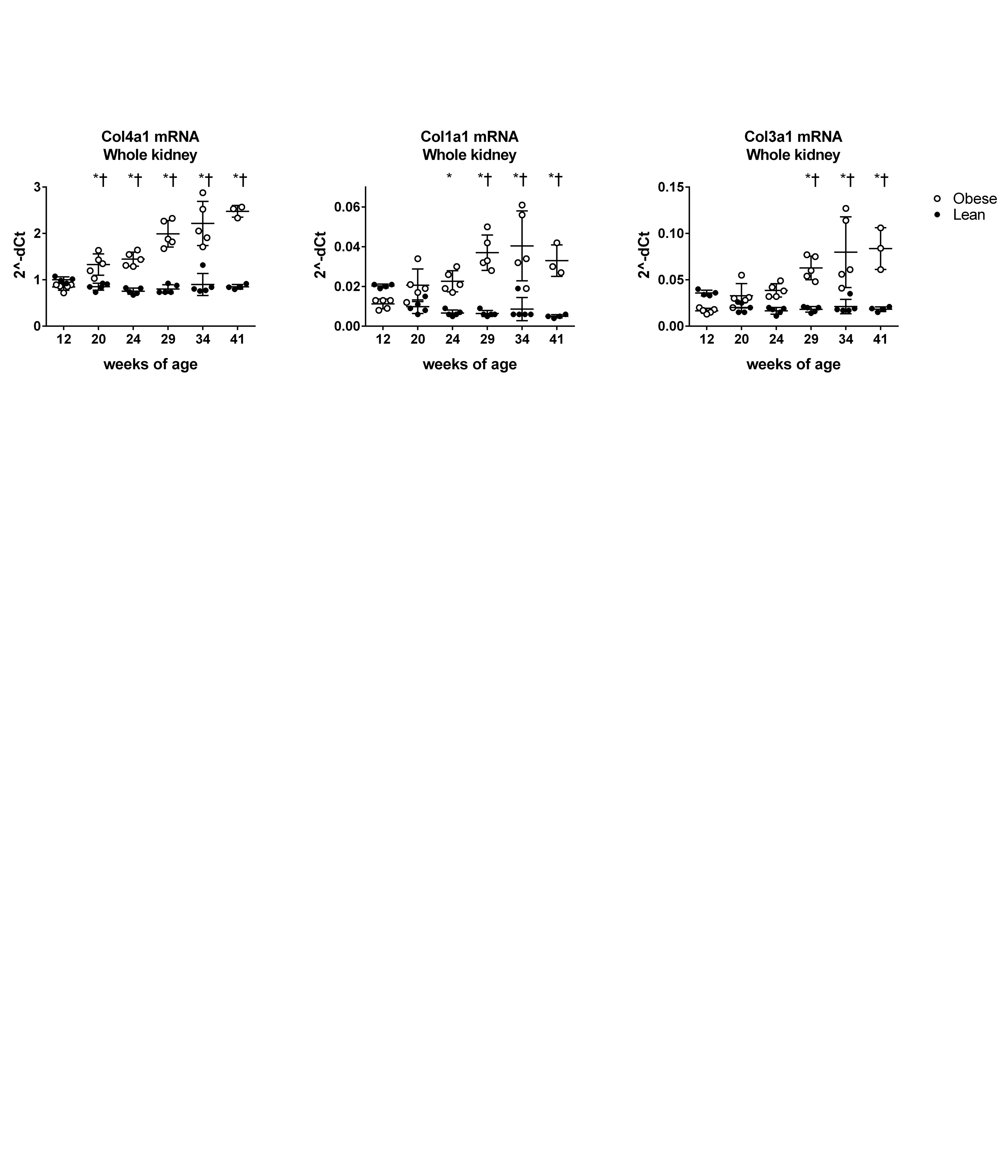

Supplement: S5 Fig — A statistical difference in the expression of these fibrotic mRNAs between age-matched lean and obese animals is evident by 19 weeks of age (Col4a1 mRNA), 24 weeks of age (Col1a1 mRNA), and 29 weeks of age (Col3a1) mRNA. Reported 2^-dCt values are versus GAPDH mRNA. (TIF) [file pone.0181861.s007.tif]

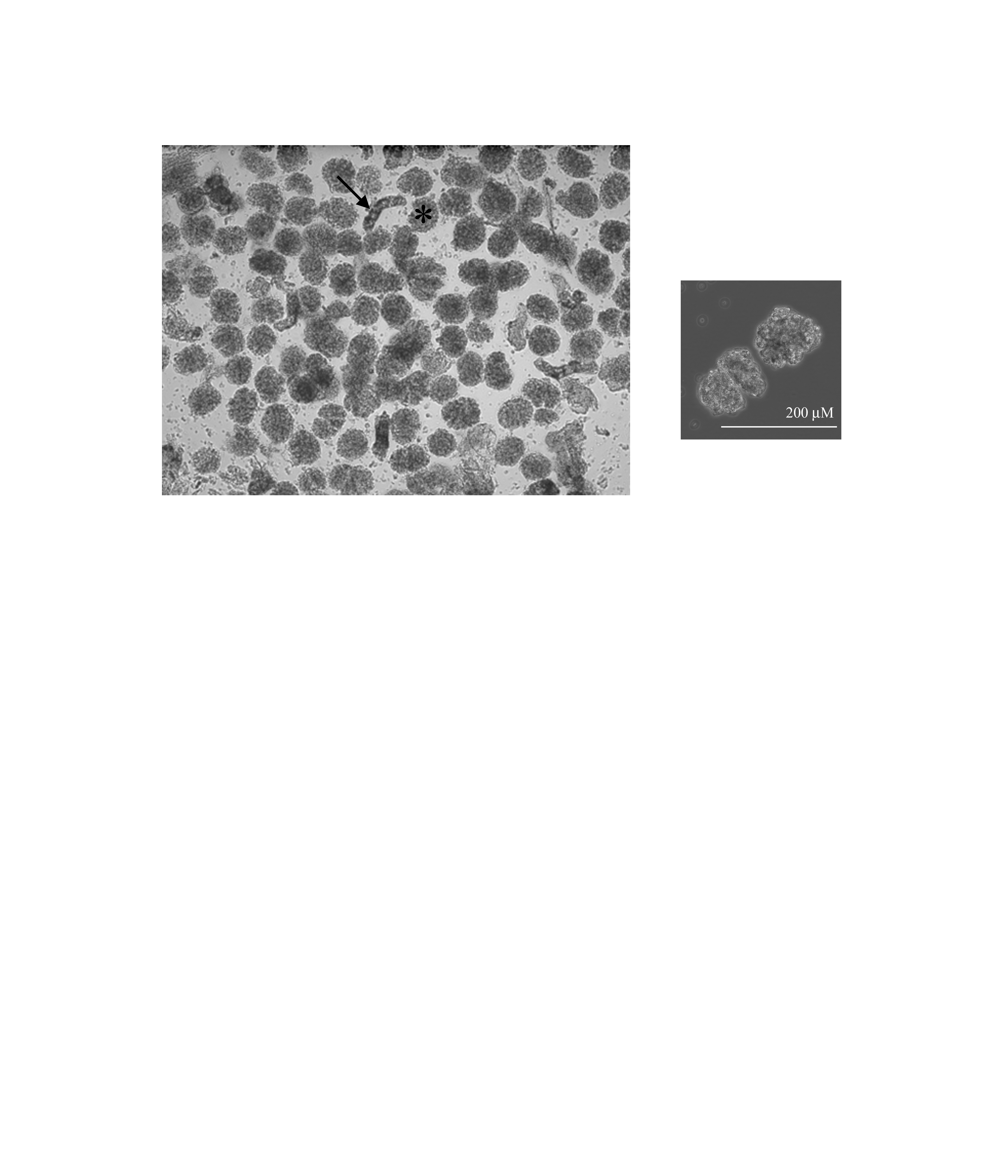

Supplement: S6 Fig — Left, the preparation is predominantly glomeruli (*), however tubule (arrow) and tubulointerstitial cells are also present. Right, higher magnification image of glomeruli isolated in this manner. Scale bar 200 μM. (TIF) [file pone.0181861.s008.tif]

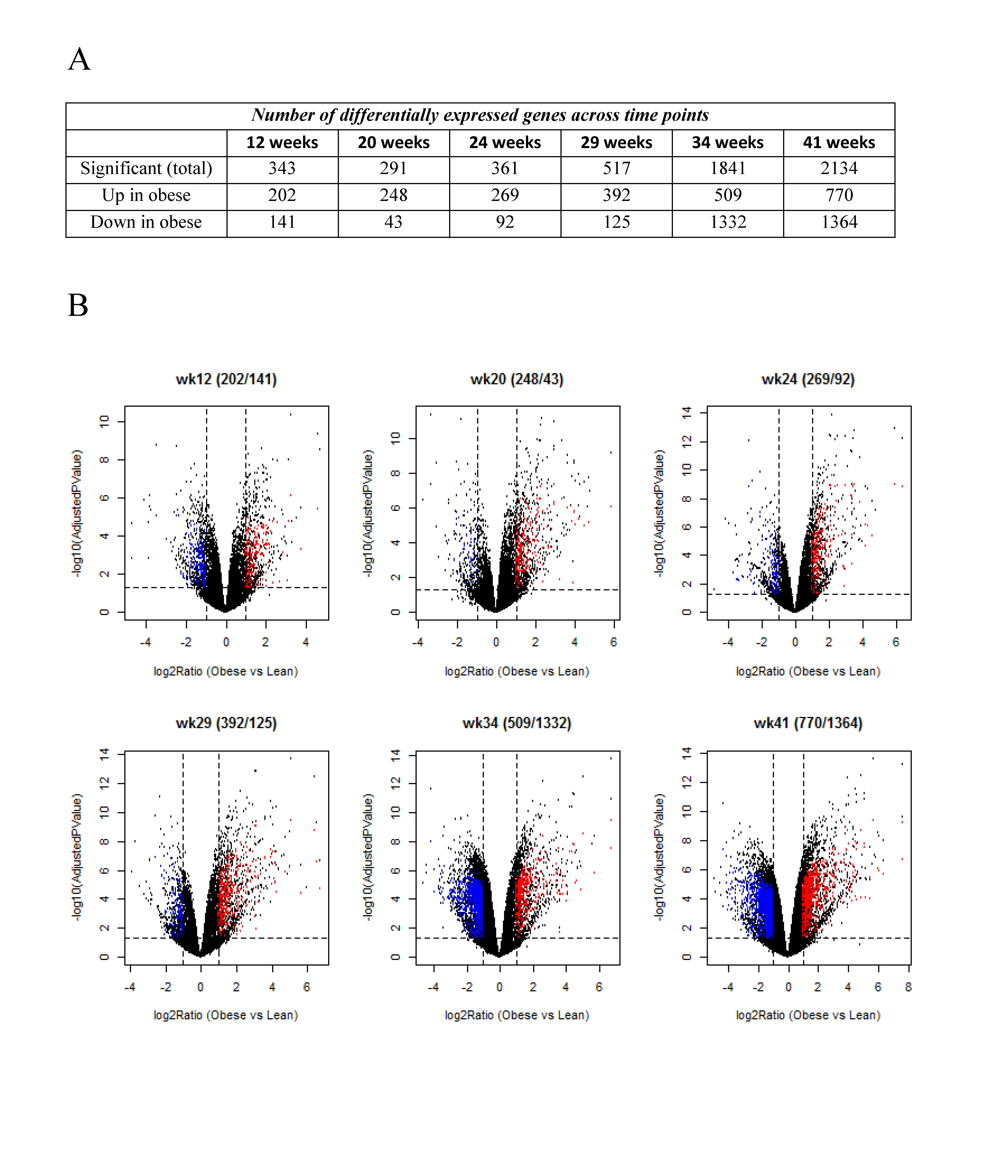

Supplement: S7 Fig — (A) The number of DEGs from a comparison of age-matched lean and obese animals trends up over time. (B) Volcano plots for DEGs. The obese group was compared with lean group, and statistically significant (FDR ≤ 0.05) up-regulated and down-regulated genes with fold change greater than 2 are colored in red and blue, respectively. (TIF) [file pone.0181861.s009.tif]

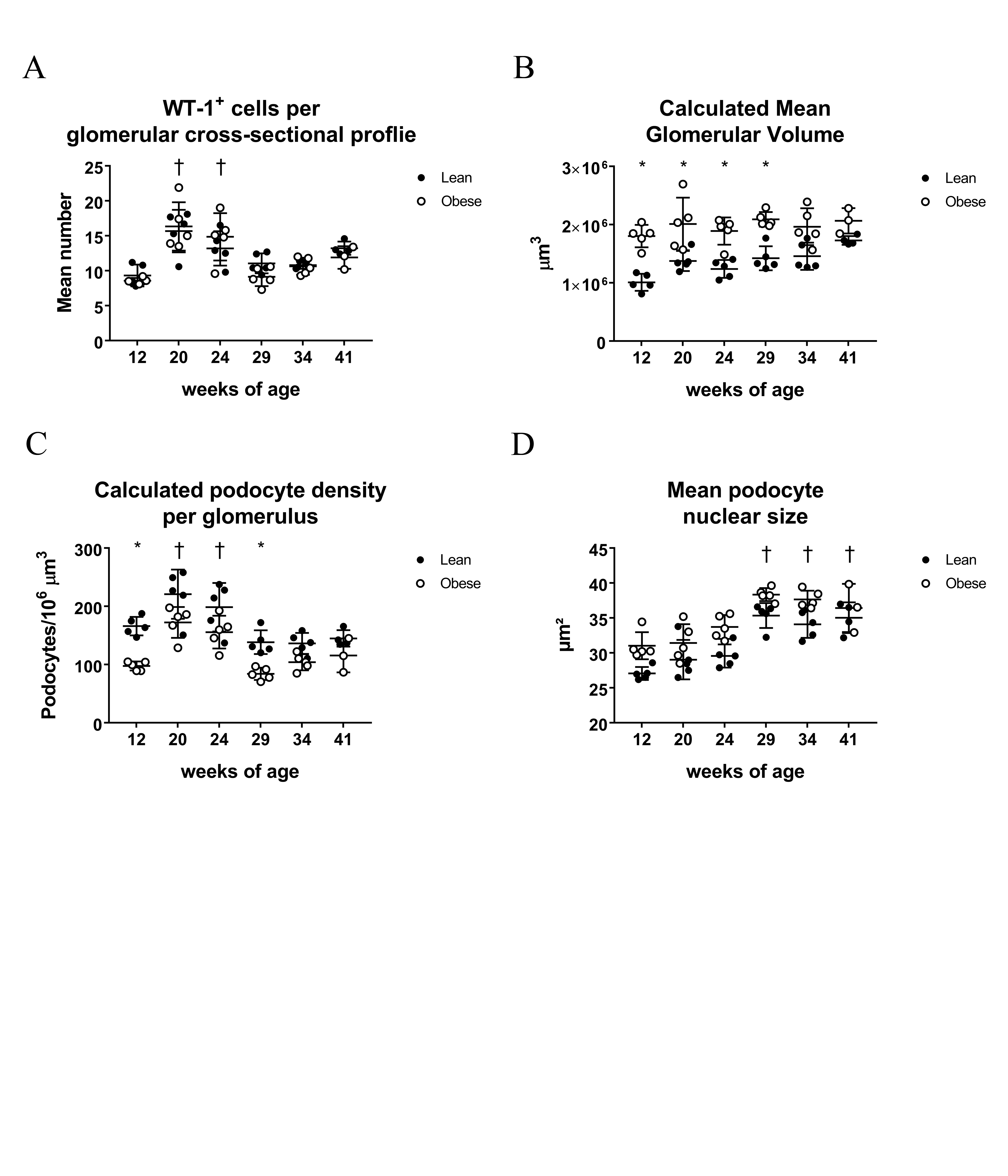

Supplement: S8 Fig — An average of 44 glomerli were analyzed for each animal. (A) Number of WT-1+ cells per glomerular cross-sectional area. (B) Mean glomerular volume (Vglom) calculated from tuft area using the Weibel-Gomez method (see Materials and Methods). (C) Estimated podocyte density per glomerulus calculated by dividing the calculated number of podocytes per glomerulus (Npod,glom; Fig 5E and Materials and Methods) by Vglom., expressed as podocytes/106 cm3. (D) Mean podocyte nuclear size, expressed as WT-1 staining area per WT-1+ cell, in μm2. (TIF) [file pone.0181861.s010.tif]

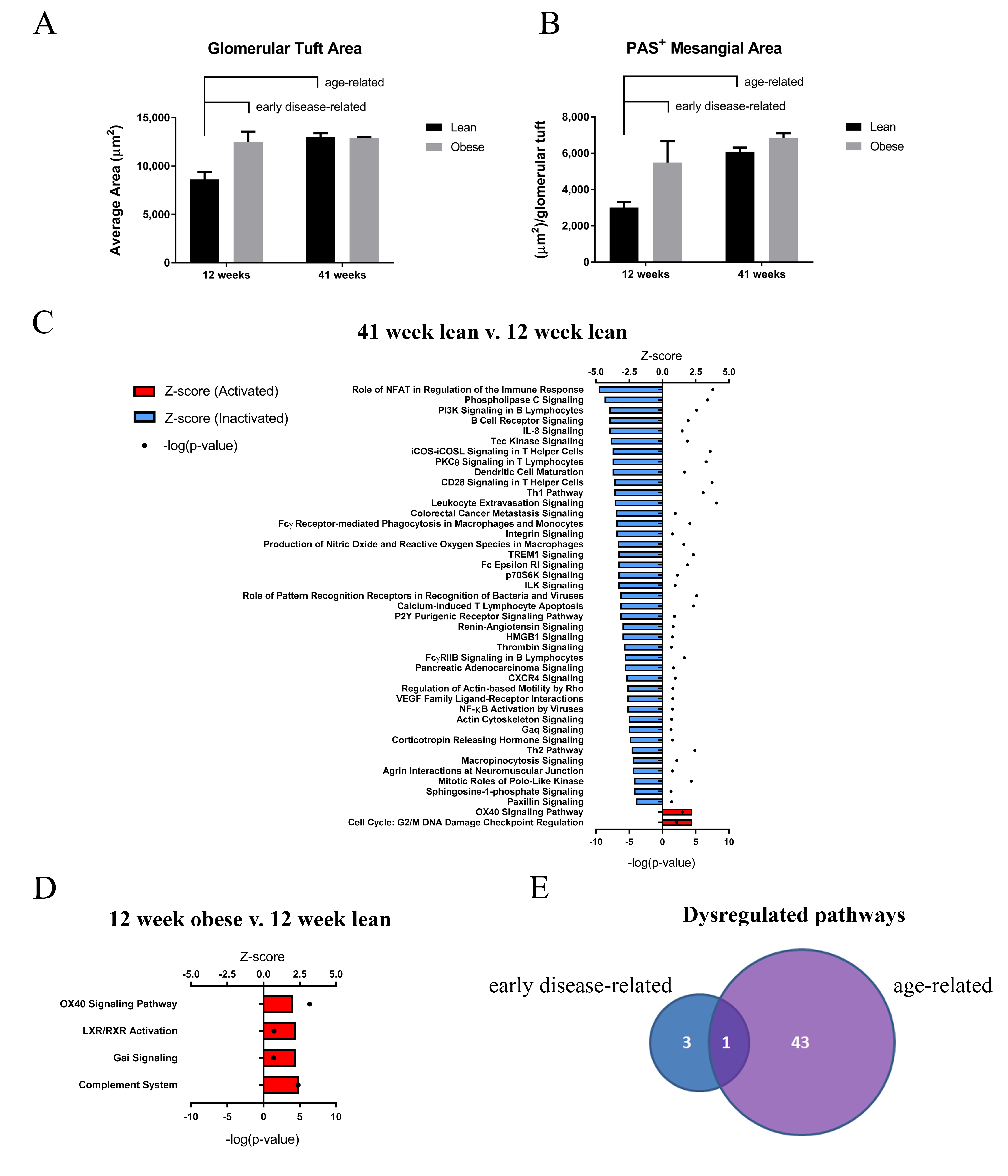

Supplement: S9 Fig — The increased glomerular tuft area (A) and PAS+ mesangial area (B) in 12-week old ZSF1 obese animals becomes obscured by an increase in both parameters in ZSF1 lean animals as they age (re-depiction of data provided in Fig 2). (C) Pathway analysis of “age-related” gene expression changes from a comparison of 41 week lean to 12 week lean animals. The bars represent the Z-score relative to the top x-axis, with activated and inactivated pathways in red and blue, respectively; the–Log(p-value) is depicted as a dot relative to the bottom x-axis scale. (D) Pathway analysis of “early disease-related” gene expression changes from a comparison of 12 week obese to 12 week lean animals. (E) Venn diagram of the 44 age-related and 4 early disease-related pathways. Only one pathway, OX40 signaling, is represented in both groups. (TIF) [file pone.0181861.s011.tif]
